# CRISPR guides induce gene silencing in plants in the absence of Cas

**DOI:** 10.1186/s13059-021-02586-7

**Published:** 2022-01-03

**Authors:** Veerendra Kumar Sharma, Sandeep Marla, Wenguang Zheng, Divya Mishra, Jun Huang, Wei Zhang, Geoffrey Preston Morris, David Edward Cook

**Affiliations:** 1 Department of Plant Pathology, Kansas State University, Manhattan, KS USA; 2 Department of Soil and Crop Science, Colorado State University, Fort Collins, CO USA

**Keywords:** CRISPR-Cas13, Virus interference, Transcript targeting, RNA silencing, Guide-induced gene silencing

## Abstract

**Background:**

RNA-targeting CRISPR-Cas can provide potential advantages over DNA editing, such as avoiding pleiotropic effects of genome editing, providing precise spatiotemporal regulation, and expanded function including antiviral immunity.

**Results:**

Here, we report the use of CRISPR-Cas13 in plants to reduce both viral and endogenous RNA. Unexpectedly, we observe that crRNA designed to guide Cas13 could, in the absence of the Cas13 protein, cause substantial reduction in RNA levels as well. We demonstrate Cas13-independent guide-induced gene silencing (GIGS) in three plant species, including stable transgenic Arabidopsis. Small RNA sequencing during GIGS identifies the production of small RNA that extend beyond the crRNA expressed sequence in samples expressing multi-guide crRNA. Additionally, we demonstrate that mismatches in guide sequences at position 10 and 11 abolish GIGS. Finally, we show that GIGS is elicited by guides that lack the Cas13 direct repeat and can extend to Cas9 designed crRNA of at least 28 base pairs, indicating that GIGS can be elicited through a variety of guide designs and is not dependent on Cas13 crRNA sequences or design.

**Conclusions:**

Collectively, our results suggest that GIGS utilizes endogenous RNAi machinery despite the fact that crRNA are unlike canonical triggers of RNAi such as miRNA, hairpins, or long double-stranded RNA. Given similar evidence of Cas13-independent silencing in an insect system, it is likely GIGS is active across many eukaryotes. Our results show that GIGS offers a novel and flexible approach to RNA reduction with potential benefits over existing technologies for crop improvement and functional genomics.

**Supplementary Information:**

The online version contains supplementary material available at 10.1186/s13059-021-02586-7.

## Background

Genome editing technologies such as CRISPR-Cas9 (clustered regularly interspaced short palindromic repeats and CRISPR-associated protein), CRISPR-Cas12, and newly identified systems enable unprecedented opportunities for genome engineering [1–4]. However, DNA editing technologies involving double-strand break repair can result in the creation of unintended DNA mutations [5, 6], potentially hindering applications. The derivative Cas9 protein, termed PRIME-editor, enables more precise editing and overcomes the unintended consequences resulting from the creation of double-strand breaks [7]. Despite these technical advances in genome engineering, there remains a potentially fundamental limitation to DNA editing, where the alteration of a gene results in unintended and unpredictable phenotypes. This will occur for genes with pleiotropic effects [8]. Additionally, many target traits for improvement are polygenic in nature, and multigene genome editing will compound the problem of generating unwanted phenotypes [9]. One approach to overcome these limitations is spatiotemporally genome editing, such as demonstrated with the CRISPR tissue-specific knockout system (CRISPR-TSKO), in which DNA is edited in specific cell types [10]. This approach will likely serve a role in future application of genome engineering, but the generation of mosaic genotypes caused by differences in the rate and penetrance of cell-specific editing, especially in polyploid crops, may limit the utility of this approach.

An alternative approach is the manipulation of RNA as it plays a central role in cellular dynamics, mediating genotype-phenotype relationship in eukaryotes. Manipulating RNA has potential advantages over DNA editing, such as circumventing negative pleiotropy, where an RNA product can be specifically spatiotemporally regulated. To manipulate complex traits, the targeting of multi-copy genes or multigene pathways through RNA manipulation offers more flexibility and precision than DNA-editing approaches. Further, RNA manipulation can also be used to target RNA viruses for engineered immunity [11]. Current RNA degradation technologies involving RNA interference (RNAi) suffer from off-target silencing [12], potentially introducing the same pleiotropic and unintended phenotypes as DNA editing.

To overcome these limitations, we sought to develop the class II type VI CRISPR-Cas13 system for use in plants, where the Cas13 nuclease specifically binds target single-stranded (ss)RNA in a CRISPR RNA (crRNA)-guided manner [13–15]. Recent reports have established the use of Cas13 as an introduced antiviral immune system in plants [16–18]. Here we report the discovery that crRNA guides alone, in the absence of Cas13, cause the reduction of both viral and endogenous plant mRNA in a sequence-dependent manner. Mechanistically, our results suggest this guide-induced gene silencing (GIGS) functions through endogenous components of the RNAi pathway and are dependent on Argonaute protein(s). The use of compact, multi-guide crRNA to elicit selective RNA reduction provides a new avenue, along with Cas13-dependent approaches, to precisely manipulate plant traits.

## Results

### crRNA guides alone, in the absence of Cas13, can elicit target RNA reduction

**Fig. 1:**
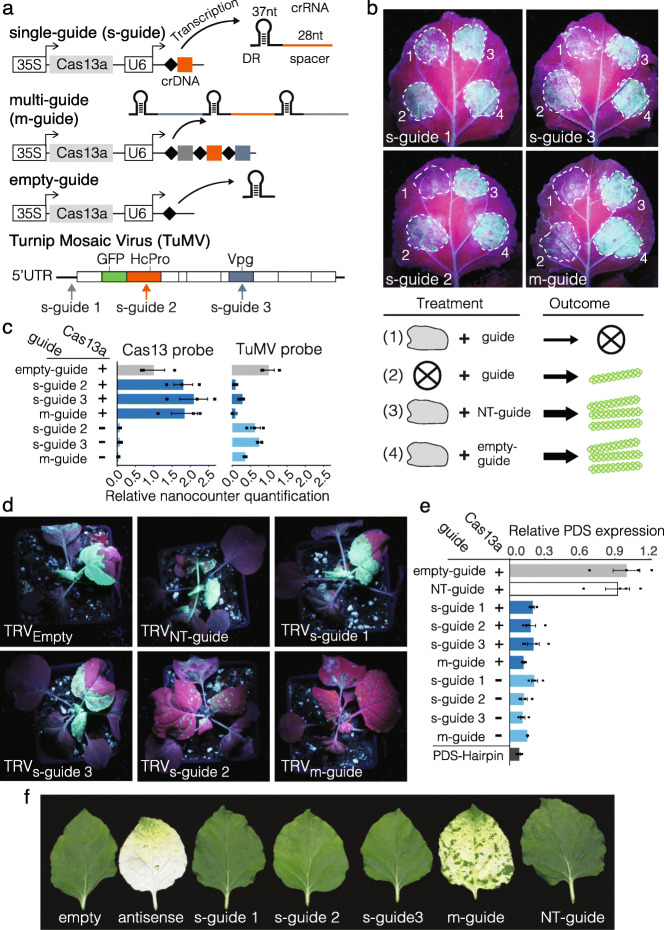
Cas13 and GIGS reduce viral and endogenous target RNA in *N. benthamiana*. **a** Schematic overview of the Cas13 transgene system. Guide crRNA responsible for RNA target specificity contain a single 28-nucleotide (nt) spacer antisense to the target RNA (single-guide, s-guide), multiple 28 nt spacers (multi-guide, m-guide), or lack the spacer (empty-guide). A diagram showing the genome of turnip mosaic virus (TuMV) expressing GFP and indicating the location of the three targeting sites for the guide crRNA. **b** The accumulation of GFP was assessed at 120 h post inoculation based on GFP fluorescence. Areas of agroinfiltration are shown in dashed white circles. Individual treatments are labeled with numbers and shown schematically below the photographs. **c** Nanostring RNA quantification for Cas13 and TuMV levels corresponding to labeled treatments for *N. benthamiana* spot infiltration. Samples expressed Cas13 (+) or not (−). **d** Representative images of *N. benthamiana* plants under UV light at 7 days post inoculation. The systemic movement of TuMV is evident based on the accumulation of GFP fluorescence for empty-guide expressing TRV (TRV_empty_). Single-guide 2 and multi-guide, TRV_s-guide 2_, and TRV_m-guide_ respectively, stopped systemic TuMV infection. **e** Quantitative PCR for the endogenous transcript *PDS* following *N. benthamiana* leaf spot infiltration. **f** Representative single leaf images of *N. benthamiana* following TRV-mediated systemic delivery of guide crRNA targeting the *PDS* transcript. Empty and non-target guides (NT-guide) did not cause photobleaching (white sectors), while the antisense and multi-guide (m-guide) did induce visible photobleaching

To test the Cas13 system in plants, we synthesized the coding sequence for two Cas13a proteins, termed LbaCas13a (from *Lachnospiraceae bacterium*) and LbuCas13a (*Leptotrichia buccalis*) for expression in plants. We tested their function *in planta* by targeting the plant infecting Turnip mosaic virus (TuMV) expressing GFP by co-expressing Cas13, crRNA targeting TuMV, and TuMV expressing GFP in *Nicotiana benthamiana* leaves using *Agrobacterium-*mediated transient expression [19, 20]. The Cas13 proteins were expressed with a single-guide crRNA containing antisense sequence to one region of the TuMV genome (single-guide), a multi-guide crRNA containing sequence against three regions of the genome (multi-guide), or an empty-guide, which contained the direct repeat (DR) crRNA sequence alone (Fig. 1a). The Cas13a protein with single- or multi-guide crRNA reduced viral accumulation, which could be observed at 72 h post inoculation (hpi) (Additional file 1: Fig. S1a). Virus accumulation was reduced by approximately 90% at 120 hpi, and TuMV interference by Cas13a was dependent on the expression of a crRNA with complementary sequence (Additional file 1: Fig. S1b-d).

In CRISPR-Cas experiments, the negative control characterizing cells expressing the sgRNA or crRNA alone, without Cas, are generally omitted due to the assumption of Cas dependence. Interestingly, we observed that expression of a single-guide or multi-guide crRNA alone, in the absence of the Cas13a protein, inhibited viral accumulation as evidenced by reduced viral genome and derived protein accumulation (Fig. 1b and Additional file 1: Fig. S2a). Viral RNA was also directly quantified using two independent NanoString nCounter probes, which allowed direct RNA quantification without the creation of complementary (c)DNA. Probes against two different regions of the TuMV genome confirmed that the single-guide and multi-guide caused virus interference when expressed with Cas13a, but also when expressed alone, in the absence of Cas13a (Fig. 1c and Additional file 1: Fig. S2b). The NanoString quantification indicated that LbuCas13a plus guides provided greater viral interference compared to the single- or multi-guide alone. Among the samples expressing guide crRNA alone, the multi-guide consistently caused the greatest TuMV reduction compared to the single-guides (Fig. 1b,c and Additional file 1: Fig. S2a,b).

To determine whether GIGS can function systemically, GIGS-mediated TuMV interference was tested using the tobacco rattle virus (TRV) expression system [21]. Plants were co-inoculated with TuMV expressing GFP and TRV, which systemically produced single- and multi-guide crRNA in the absence of Cas13 (Additional file 1: Fig. S3a). At 7 days post inoculation (dpi), GFP fluorescence from TuMV was observed in the upper systemic leaves of plants co-inoculated with either TRV expressing an empty-guide or a non-targeting (NT)-guide, which showed that systemic TRV delivery alone did not interfere with TuMV replication, movement, or translation (Fig. 1d). Samples expressing the two single-guides, s-guide 1 and s-guide 3, also accumulated visible GFP fluorescence in upper, non-inoculated leaves, indicating the spread of TuMV. Interestingly however, TRV expressing either single-guide 2 or the multi-guide caused a significant reduction in GFP fluorescence in the upper systemic leaves (Fig. 1d, and Additional file 1: Fig. S3b). Quantitative assessment of TuMV accumulation in systemic leaves by qPCR showed an approximately 90% reduction in TuMV accumulation in samples expressing single-guide 2 and the multi-guide (i.e., GIGS) (Additional file 1: Fig. S3c). Moreover, qPCR revealed an approximate 30 to 40% reduction in TuMV levels when TRV expressed single-guide 1 or single-guide 3, which was not obvious from visual inspection of GFP fluorescence. This may reflect complicated translation mechanisms viruses employ, such as internal ribosome entry [22], in which the viral molecule was targeted by GIGS and partially interfered with, while intact GFP open reading frame sequence was still translated. These results indicate that GIGS can cause systemic TuMV interference, but that crRNA target sequences vary in effectiveness. Variation for crRNA effectiveness has been reported for Cas13-dependent RNA targeting, likely caused by secondary structure and accessibility of the target RNA [23].

Viruses manipulate host physiology and have unique features unlike host-derived RNAs [24, 25], making it possible that the GIGS phenomena is limited to viral RNA. To test this hypothesis, we targeted endogenous phytoene desaturase (*PDS*) mRNA with single-guide and multi-guide crRNA with and without LbuCas13a (Additional file 1: Fig. S4). *Agrobacterium*-mediated expression of single- and multi-guide crRNA with and without LbuCas13 caused a significant reduction in *PDS* transcript levels compared to expressing LbuCas13a alone or with a NT-guide (Fig. 1e). The resulting mRNA reduction (75–85%) was consistent across the tested samples, comparable to a PDS hairpin construct known to induce RNAi (Fig. 1e). The reduction in *PDS* mRNA was confirmed by northern blot, which showed a clear reduction for *PDS* signal for both LbuCas13a-dependent and GIGS compared to expressing LbuCas13a alone, with a NT-guide, or from an untreated leaf (Additional file 1: Fig. S5a). Direct RNA quantification by NanoString further confirmed a significant reduction for the *PDS* transcript for samples expressing the *PDS* targeting guides with or without the expression of Cas13a (Additional file 1: Fig. S5b). These results establish that GIGS acts on both viral RNA and endogenous transcripts.

To test if GIGS acts systemically on endogenous genes, TRV expressing guides targeting endogenous *PDS* mRNA were infiltrated into *N. benthamiana* (Additional file 1: Fig. S6). Under the hypothesis that GIGS can act systemically on endogenous genes, the prediction is that TRV-delivered guides result in photobleaching in TRV-infected tissues. Three single-guide crRNA, targeting different regions of *PDS*, did not exhibit significant photobleaching (Fig. 1f). However, two multi-guides targeting different *PDS* regions displayed substantial photobleaching in systemic leaf tissue (Fig. 1f and Additional file 1: Fig. S7a). Interestingly, the visible photobleaching pattern induced by the antisense fragment (i.e., RNAi) and that induced by GIGS were not the same (Fig. 1f and Additional file 1: Fig. S7a). While the antisense RNAi photobleaching was strong in the upper, youngest leaves, GIGS-induced photobleaching was not visible in the upper most leaves, and the photobleaching occurred in more distinct segments causing a patchy appearance. Quantifying the photobleaching to confirm the phenomena, SPAD meter readings showed a significant reduction in chlorophyll content for samples expressing the multi-guide crRNAs and containing the antisense *PDS* fragment (Additional file 1: Fig. S7b). Plants that expressed single-guide 2 were yellow and also showed a reduced SPAD reading (Additional file 1: Fig. S7a,b). Quantifying *PDS* transcripts with qPCR showed that the *PDS* transcript level was reduced (30–45%) for the three single-guides, and to a greater extent by the multi-guides (65–70%) and the antisense construct (85%) (Additional file 1: Fig. S7c). It is not clear why single-guide 1 and 3 caused a reduction in *PDS* mRNA levels, but did not result in visible photobleaching or SPAD meter reductions, but we note that the reduced *PDS* mRNA levels are consistent with that seen using *Agrobacterium*-mediated spot infiltration (e.g., Fig. 1e and Additional file 1: Fig. S5). Collectively, we found that GIGS induced by multi-guides caused a greater reduction in target transcript levels compared to that induced by single-guides for both virus and endogenous RNA targeting.

### GIGS functions in multiple plant species and is heritable in *Arabidopsis*

**Fig. 2:**
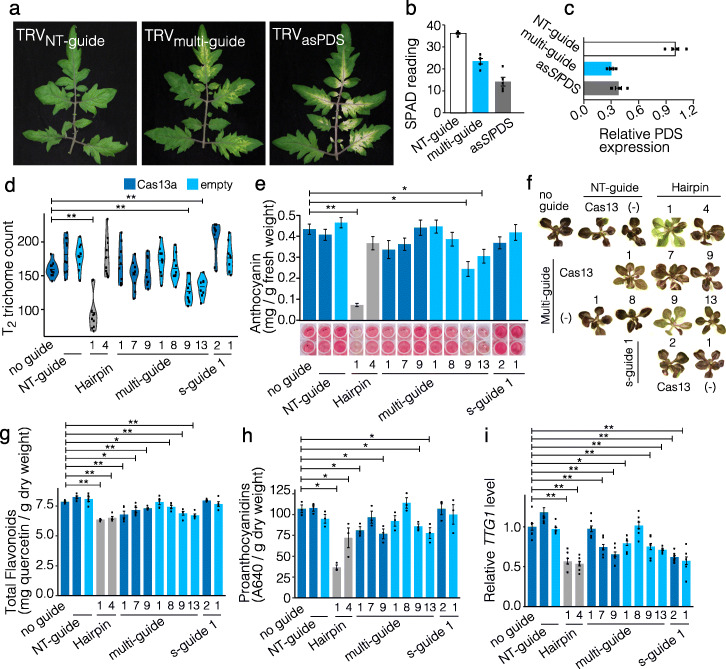
Cas13 and GIGS function across plant species and are heritable. **a** Representative images of tomato leaves following TRV systemic movement and photobleaching induced by GIGS (TRV_m-guide_) and an antisense transcript (TRV_asPDS_). TRV expressing a non-targeting guide crRNA (TRV_NT-guide_) does not induce photobleaching. **b** Measurements of chlorophyll content from SPAD meter readings for three independent plants. SPAD meter readings were taken from leaf sections showing photobleaching, and individual reading are shown as black points with the mean and standard deviation shown as a bar plot. **c** qPCR measurement of the *PDS* transcript standardized to the *EF1α* transcript and relative to the NT-guide sample. Three independent samples were analyzed, and individual data are shown as black points with the mean and standard deviation shown as bar plots. **d–i** Data for independent transgenic *Arabidopsis* lines. Data for plants expressing LbuCas13a are shown in dark blue and plants not expressing the protein are shown in light blue. Control lines expressing a hairpin construct against the *TTG1* transcript are shown in gray. **d** Trichome counts from the seventh leaf of T_2_ *Arabidopsis* lines. Ten plants were counted per independent line, listed below graph, with the individual counts shown as black points and the distribution represented as a violin plot. **e** Leaf anthocyanin quantification from T_3_ seedlings following sucrose treatment. Representative wells following extraction shown below each bar plot. **f** Representative plantlets following sucrose treatment showing anthocyanin pigmentation (i.e., purple color). **g** Total flavonoids extracted from seeds collected from T_2_ plants. Five independent seed lots were analyzed per line, shown as black points. **h** Seed proanthocyanidin quantification from the same plants analyzed in **g**. **i** Quantification of the *TTG1* transcript from three T_2_ and three T_3_ plants per line, individual data shown as black points. **d,e,g–i** Statistical comparisons were made between the transformation control (no guide) and each treatment using a one-sided Mann-Whitney *U* test with Benjamini-Hochberg (BH) multiple testing correction. A line is shown above each pair of bars to indicate each pairwise comparison that had a statistically significant difference. This is indicated for comparisons where the adjusted *p* value was less than 0.05 (*) and 0.01 (**)

An important question is whether GIGS is limited to *N. benthamiana* or is more broadly active in plants. To test this, multi-guide crRNA were developed to target *PDS* in tomato (*Solanum lycopersicum*), which were delivered using TRV, along with a NT-guide and an antisense *PDS* control. We observed visible photobleaching in upper leaves of *S. lycopersicum* plants following systemic movement of TRV expressing a multi-guide-targeting *S. lycopersicum PDS*, although the photobleaching was not as widespread as that produced by the antisense *PDS* construct (Fig. 2a). Quantifying chlorophyll levels and the *PDS* transcript indicated that photobleached tissue from GIGS and antisense expressing TRV both had substantially lower levels compared to the control (Fig. 2b,c). These results show that GIGS is active outside of *N. benthamiana*, possibly extending to other plants in the *Solanaceae* family.

Another important question is whether GIGS requires bacterial or viral machinery (i.e., proteins) introduced during transient expression or if GIGS functions in stable transgenics through plant endogenous machinery. To test these hypotheses, and further test the generality of GIGS in plants, we transformed *Arabidopsis thaliana* (Col-0) with single-guide and multi-guide crRNA targeting the pleiotropic regulatory gene *TRANSPARENT TESTA GLABRA1* (*TTG1*), both with and without LbuCas13a. The *TTG1* gene encodes a WD40 repeat protein, which interacts with MYB and bHLH transcription factors required for normal trichome and root hair development, along with seed proanthocyanidin and vegetative anthocyanin production [26–28]. The average trichome counts for multiple independent T_1_ plants that expressed LbuCas13a with either single-guide or multi-guide crRNA had significantly fewer trichomes compared to wild-type, and importantly, plants expressing single-guides and the multi-guide crRNA, without Cas13, also had significantly fewer trichomes on average (Additional file 1: Fig. S8a). The *TTG1* transcript was quantified in T_1_ plants and was highly variable across the transformed lines (Additional file 1: Fig. S8b). Individual plants were selected and self-fertilized, and seeds from T_1_ plants showed reduced total flavonoids in both Cas13 and GIGS lines, consistent with reduced *TTG1* (Additional file 1: Fig. S8c).

We assessed whether GIGS would function in progeny inheriting guides by characterizing individual lines in the T_2_ and T_3_ generations for alteration of *TTG1*-dependent phenotypes. Trichome counts of the seventh leaf (from ten plants per line) indicated that two GIGS lines (i.e., expressing only a multi-guide crRNA targeting *TTG1*), and one of the hairpin expressing lines had significantly fewer trichomes compared to the transformation control expressing Cas13a alone (Fig. 2d). Individual transformed lines were subjected to sucrose and light stress to induce leaf anthocyanin production, and we again observed that two lines expressing multi-guide crRNA targeting *TTG1* (i.e., GIGS) displayed significantly reduced leaf anthocyanin levels, along with a hairpin expressing line (Fig. 2e,f). Quantification of total seed flavonoids showed a significant but modest reduction compared to the control line, for both Cas13 expressing and GIGS lines along with both hairpin expressing lines (Fig. 2g). Total flavonoid quantification also measures products upstream of *TTG1* regulation, which can confound the impact of *TTG1* reduction. To more accurately assess the impact of *TTG1* reduction, we measured seed proanthocyanidins, which are controlled downstream of *TTG1*. This analysis identified a more substantial impact for *TTG1* reduction, where the level of proanthocyanidins were significantly reduced (Fig. 2h), and were consistent with the results from the total flavonoid quantification (Fig. 2g).

These results indicate heritable phenotypes for multiple traits mediated by both Cas13 and GIGS in stable transgenic *Arabidopsis* when targeting the pleiotropic regulator *TTG1*. We do note there was substantial phenotypic variation among lines with the same construct, despite significant reduction in *TTG1* levels (Fig. 2i). This is in part explained by variation in transgene expression and translation (Additional file 1: Fig. S9). In addition, there are more complicated mechanisms that may explain the results, such as asynchronous *TTG1* expression and Cas13 or GIGS expression at the individual cell level, or the effect of incomplete *TTG1* silencing on trait manifestation (i.e., kinetics of silencing to produce a phenotype) [29, 30]. Optimizing Cas13 and GIGS approaches will be an important step to deliver robust biotechnology platforms for plant research and crop improvement, particularly for tissue- or temporal-specific expression that is difficult to manipulate precisely with CRISPR-Cas9.

### Multi-guide crRNA induce secondary small RNA production

**Fig. 3:**
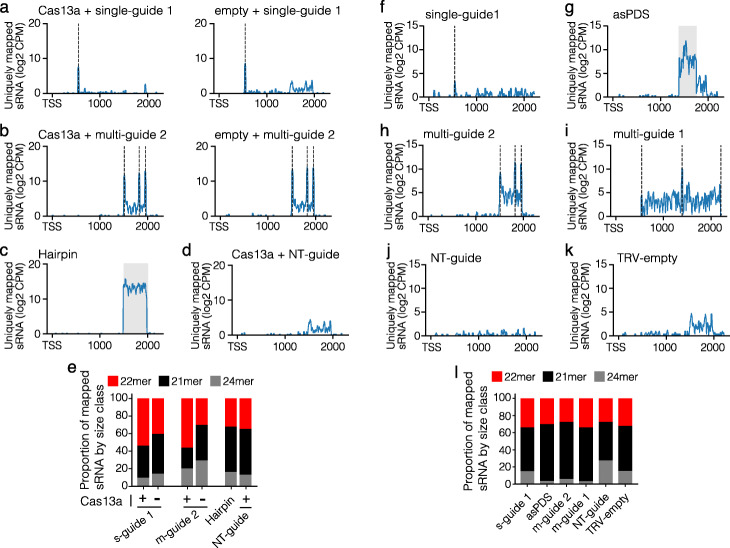
Multi-guide-induced GIGS results in sRNA generation. **a–d** Uniquely mapped small RNA (sRNA) read counts to the *PDS* transcript collected five days post agro-mediated spot infiltration. Read counts are log_2_ of counts per million + 1 (log_2_ CPM) and shown relative to the transcription start site (TSS) till the end of the predicted mRNA (2216 bp). Individual treatments are labeled above each graph, and one of the two replicate samples per treatment is plotted. The positions of the expressed single- and multi-guide crRNA are shown as vertical dashed line(s). The region spanning the hairpin construct is shown as a gray window. **e** Proportion of 21-, 22-, and 24-nt sRNA mapped to the *PDS* transcript averaged between the two replicates. **f–l** Similar layout as described in **a–e** but here RNA was collected from systemic leaves 2 weeks following TRV expression. The treatments are listed above each graph

We sought to understand the mechanism giving rise to GIGS (i.e., guide crRNA reducing viral and endogenous RNA levels). Given that crRNA are composed of short antisense sequences, it is possible that GIGS functions through components of the endogenous RNA interference (RNAi) pathway. However, the structure of crRNA used here are not similar to hairpin RNA, small interfering RNA (siRNA), or microRNA (miRNA); therefore, it is not obvious how crRNA might enter or induce RNAi [31, 32]. Alternatively, it is possible that GIGS elicits other endogenous endo- or exonucleolytic RNA degradation pathways [33]. Since small RNA (sRNA) usually in the range of 21 to 24 nucleotides (nt) are a hallmark for RNAi, we reasoned that if GIGS functions through RNAi, abundant sRNA should be observed [34]. To assess this, we conducted small (s)RNA-seq from *N. benthamiana* samples expressing single- and multi-guide crRNA against the endogenous *PDS* transcript. The sRNA mapped to the expressed crRNA indicated that the crRNA were expressed (Additional file 1: Fig. S10). For samples that expressed a single-guide, uniquely mapped sRNA showed a single sharp peak at the *PDS* transcript, which corresponds to the location of the crRNA guide sequence, regardless of Cas13 expression (Fig. 3a). The samples expressing the multi-guide crRNA had three distinct peaks of mapped sRNA, each corresponding to the location of the targeting guide sequence. However, in these samples, we also identified many sRNA mapping to the *PDS* transcript that were independent from the multi-guide target sequence (Fig. 3b). Interestingly, these sRNA were identified only between the 5′ and 3′ boundaries of crRNA targeting sites and do not appear to extend past this region (Fig. 3b). This was similar to the sRNA mapping from the samples expressing the *PDS* hairpin, which produced ample sRNA between the two ends of the hairpin fragment (Fig. 3c). While the most abundant peaks for the multi-guide crRNA samples corresponded to the guide targets themselves, the identification of thousands of sRNA reads between these target regions suggest the production of secondary sRNA. We do note the presence of background sRNA in the samples where Cas13 was expressed with a NT-guide, which may indicate background read mapping or potentially RNA contamination during library preparation, but the signal was low (Fig. 3d). Supporting the idea that GIGS results in the production of secondary sRNA through RNAi, we identified more 21 nt sRNA (i.e., siRNA) mapped to the *PDS* transcript during GIGS (i.e., without the Cas13 protein) than when Cas13 was expressed with the guide (Fig. 3e).

To further determine sRNA production during GIGS, a second sRNA-seq experiment was conducted by expressing either a single-guide or one of two multi-guide crRNA in the absence of Cas13 using the TRV vector in *N. benthamiana*. The sRNA mapped to the expressed crRNA again indicated expression of the crRNA (Additional file 1: Fig. S11). For samples expressing the single-guide, there was a clear but small peak of uniquely mapped sRNA corresponding to the guide target sequence, along with other background mapped sRNA (Fig. 3f). In contrast, mapped sRNA from the sample expressing a *PDS* antisense fragment produced many sRNA targeting the PDS transcript and mostly correspond to the sequence that was expressed in the antisense fragment (Fig. 3g). Both multi-guide crRNAs showed three sharp peaks of mapped sRNA, with each peak corresponding to a guide-targeting region (Fig. 3h,i). Importantly, these samples clearly have many mapped sRNA that are outside of the multi-guide targeted region, which are not present in the controls, and were not expressed as part of the multi-guide crRNA sequence (Fig. 3 h–k). We interpret these sRNA to represent secondary sRNA generated in response to multi-guide GIGS. Consistent with these secondary sRNA being generated via components of the RNAi pathway, the length of sRNA mapped to the *PDS* transcript are predominantly 21 nt for the two multi-guide and antisense fragment samples (Fig. 3l). These results suggest that siRNA and RNAi are likely involved in mediating GIGS.

### GIGS RNA reduction may function through Argonaute

**Fig. 4:**
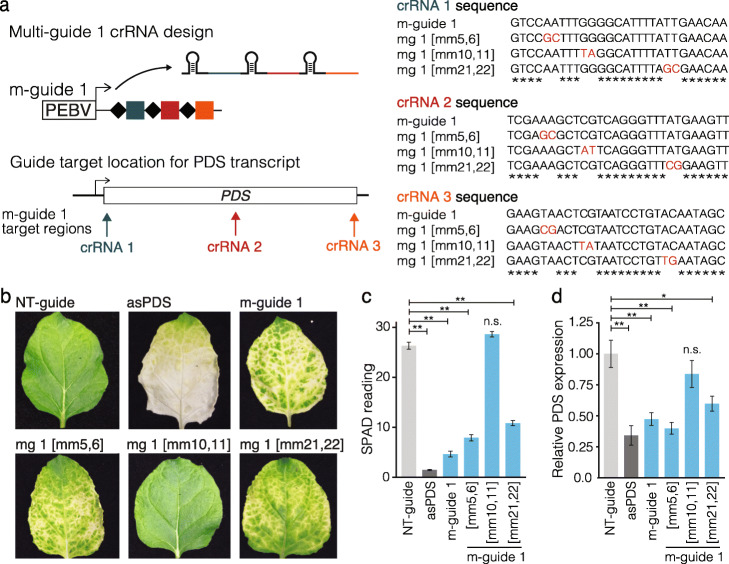
Guide mismatches at position 10 and 11 abolish GIGS, indicating AGO dependence. **a** Illustration of multi-guide expression from TRV targeting the *PDS* transcript. For each of the 28-nt guides (crRNA1, crRNA2, crRNA 3) a variant m-guide 1 was designed. For mg 1[mm5,6], each crRNA contained two base pair mismatches at positions 5,6, for mg 1[mm10,11] mismatches at positions 10,11, and mg 1[mm21,22] contained mismatches at positions 21,22. **b** Representative images of leaves following TRV systemic delivery of m-guide 1 targeting *PDS*, in addition to the three variants of m-guide 1. TRV expressing a non-targeting guide (NT-guide) and TRV with a region of antisense sequence to *PDS* (asPDS) served as controls. **c** SPAD meter readings from photobleached (loss of green color) leaf samples. Data collected from a total of six independent leaves from two experiments. **d** Quantification of the *PDS* transcript using qPCR for the same samples as measured in **c**. Data standardized to an endogenous transcript and normalized to TRV expressing NT-guide. Statistical comparisons were made between the NT-guide and each treatment using a one-sided Mann-Whitney *U* test with Benjamini-Hochberg (BH) multiple testing correction. Samples with *p* values less than 0.05 (*) and 0.01 (**) are indicated. n.s., non-significant difference (*p* > 0.05)

Under the hypothesis that GIGS requires endogenous RNAi machinery, target mRNA reduction would be dependent on Argonaute (AGO) RNA-binding protein(s) [35]. AGO proteins are required to form the RNA Induced Silencing Complex (RISC), which carries out the biochemical slicing or translational inhibition of target mRNA [36, 37]. To achieve AGO-mediated endonuclease activity, perfect complementary base pairing is required at positions 10 and 11 of the AGO-bound siRNA with the target mRNA (i.e., central duplex region) [38–40]. Therefore, if GIGS is dependent on AGO, multi-guide crRNA designed to have mismatches at base pairs 10 and 11 should be blocked for GIGS (i.e., no target mRNA reduction). To test this, multi-guide crRNA that contained specific two base pair mismatches to the *PDS* mRNA were delivered to *N. benthamiana* using TRV (Fig. 4a). The results showed that multi-guide crRNA against *PDS* with mismatches at the critical region for AGO endonuclease activity (i.e., base pairs 10,11) did not cause photobleaching, while negative control mismatches (i.e., positions 5,6 or 21,22) still elicited photobleaching (Fig. 4a, Additional file 1: Fig. S12 for whole plant images). The chlorophyll content as measured by SPAD meter was not significantly different between the NT-guide control and the multi-guide with mismatches at positions 10,11 (mg 1[mm10,11]) (Fig. 4c). The perfect complementary multi-guide, along with the guide containing mismatches at positions 5,6 and 21,22 had significantly reduced SPAD meter readings, along with the antisense *PDS* construct (Fig. 4c). Quantification of *PDS* transcripts by qPCR confirmed no reduction for samples expressing the multi-guide with position 10,11 mismatches, while all other treatments significantly reduced the level of the *PDS* transcript (Fig. 4d). We note that the mismatches at 5,6 and 21,22 did affect silencing, as the perfectly complementary multi-guide crRNA gave the strongest photobleaching. These mismatches may interfere with other RISC functions, such as target recognition and target mRNA turnover [38, 40]. However, it is clear that mismatches at 10,11 abolish GIGS, while the other mismatches diminish it, suggesting that GIGS functions through one or more endogenous AGO proteins. Additionally, these results suggest that GIGS is mediated by RNA endonuclease reduction and not translational inhibition of target mRNA [41].

### Guides do not require Cas13 crRNA sequence to elicit GIGS

The Cas13 guide crRNA are composed of the Cas13 specific direct repeat (DR) domain and the antisense target sequence [42], and they do not contain double-stranded RNA corresponding to the target sequence as would be found in a hairpin, short-hairpin or miRNA transgene. It was therefore not clear if a sequence or structure of Cas13 designed crRNA were required to elicit GIGS. It was recently reported that crRNA guides from the Cas13b system cause target mRNA reduction in the absence of Cas13b, termed Cas13b-independent silencing in mosquito [43]. That report does not provide functional data that elucidate the mechanism, but the authors postulate that Cas13b-independent silencing is related to RNAi. Importantly, the Cas13b DR sequence is different than the Cas13a DR sequence used here. Additionally, the structure of the crRNA are different, where the Cas13b DR is located at the 3′ end of the crRNA following the target guide sequence, while the Cas13a crRNA used here have a 5′ DR prior to the target sequence [42]. These results suggest that GIGS is not dependent on a specific Cas13 DR sequence or structure.

**Fig. 5:**
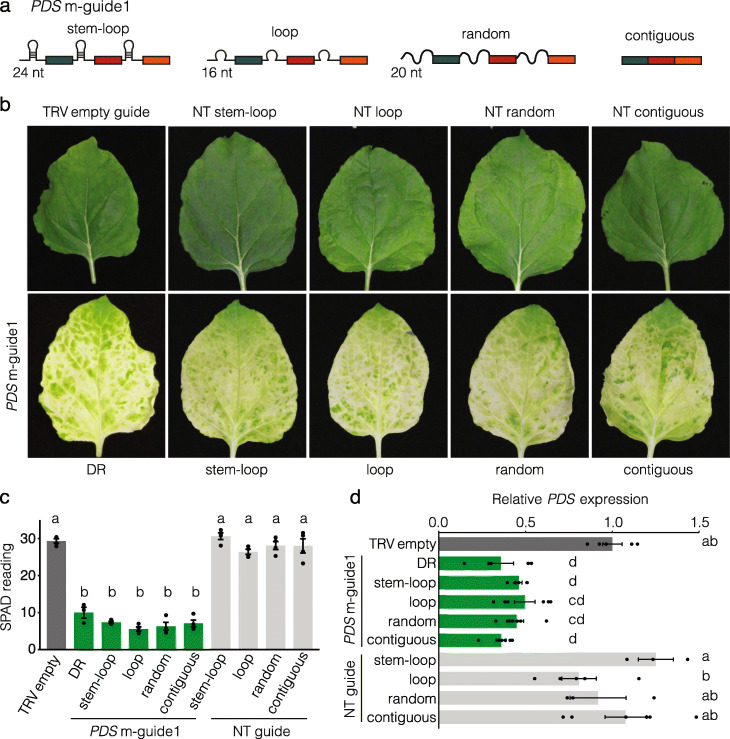
The Cas13 DR sequence is not required to elicit GIGS. **a** Schematic representation of modified *PDS* m-guide1 sequences where the LbuCas13a DR was replaced with Arabidopsis miR170 stem-loop (stem-loop, 24 nt), the miR170 without the four base pair stem (loop, 16 nt), a random sequence (random, 20 nt), and without any intervening sequence (contiguous). **b** Representative images of *N. benthamiana* leaves after 2 weeks of TRV systemic delivery. The guide delivered for each TRV construct is labeled above or below the image. The bottom row shows leaves that all expressed a version of PDS m-guide 1. **c** Chlorophyll content (leaf greenness) measured with SPAD meter 2 weeks after systemic delivery of crRNA. **d** Relative *PDS* transcript levels quantified using qPCR, compared between the infiltration control (TRV_empty_) and each treatment. Statistical comparisons were made using one-way ANOVA with multiple comparisons of treatments by means of Tukey performed using honestly significant difference. Treatments with the same letter are not significantly different

To directly investigate this hypothesis, we tested if GIGS was elicited using alternative guide designs. We constructed four new multi-guide constructs where the Cas13 DR sequence was replaced with the stem-loop sequence of *A. thaliana* miRNA170, the miRNA170 loop sequence, a random 20 nt sequence, or no intervening sequence between the guide spacer sequences (Fig. 5a). For each guide design, a control multi-guide was constructed with the same design parameters, but expressing a non-targeting guide sequence, and all constructs were delivered to *N. benthamiana* plants using the TRV delivery system. All four new guide designs targeting the *PDS* transcript caused visible photobleaching in upper systemic leaves, similar in appearance to the originally tested Cas13 m-guide containing the DR sequence, which was confirmed based on SPAD meter reading (Fig. 5b,c). The paired designs containing the NT sequence did not cause obvious phenotypes or a reduction in chlorophyll content (Fig. 5b,c). Quantifying the *PDS* transcript with qPCR showed that the four new guide designs caused similar levels of transcript reduction compared to the Cas13 designed crRNA, all of which were significantly reduced compared to the controls (Fig. 5d).

### Cas9 designed sgRNA with longer guide sequence can elicit GIGS

**Fig. 6:**
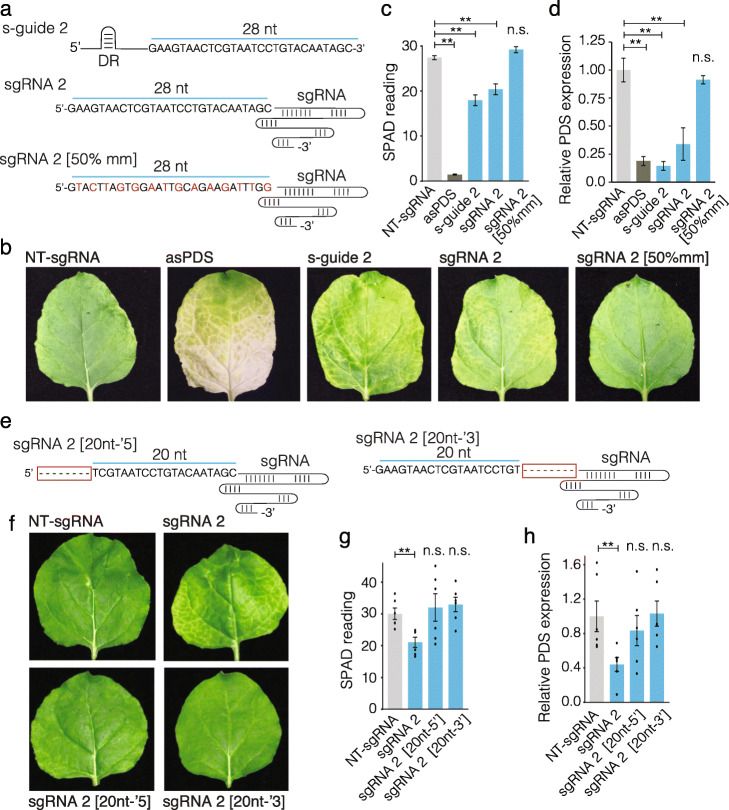
GIGS is also evident for sgRNA guides designed for Cas9. **a** Schematic of guide designs targeting *PDS* transcript for Cas13 s-guide 2, and Cas9 sgRNA 2. Each guide contains 28 nt antisense to the *PDS* transcript (sequence shown). The Cas9 sgRNA control contained 50% mismatch sequence to the *PDS* transcript (sgRNA 2 [50% mm]). Mismatch nucleotides are colored red, while shared nucleotides between the three guides are black. The Cas13 crRNA contains the 37-bp direct repeat (DR) sequence at the 5′ end. The Cas9 sgRNA contains the 78 bp trans-activating crRNA (tracrRNA, depicted as a line) at the 3′ end. **b** Representative images of leaves following TRV systemic delivery of single-guide 2 (s-guide 2) targeting *PDS*, and a Cas9 designed sgRNA designed to contain the same 28 bp targeting PDS as in s-guide 2. An sgRNA 2 control contained the sequence in sgRNA 2, but with 50% mismatches to the *PDS* transcript (sgRNA 2[50%mm]). Control sgRNA containing non-targeting guide sequence (NT-sgRNA). Photobleaching is seen in the asPDS sample, while interveinal yellowing is visible in the samples expressing s-guide 2 and sgRNA 2. **c** SPAD meter readings from photobleached leaf samples as described for **b**. Data collected from a total of six independent leaves from two experiments. **d** Quantification of the *PDS* transcript using qPCR for the same samples as measured in **c**. Data standardized to an endogenous transcript and normalized to TRV expressing NT-sgRNA. **e** Schematic of the modified sgRNA 2 targeting PDS where 8 nt were removed from the 5′ or 3′ end respectively, indicated by dashes in a red box. **f** Representative images of leaves following TRV systemic delivery of the indicated guides. **g** SPAD meter readings from photobleached leaf samples. **h** Quantification of the *PDS* transcript using qPCR for the same samples as measured in **g**. Statistical comparisons were made between NT-sgRNA and each treatment using a one-sided Mann-Whitney *U* test with Benjamini-Hochberg (BH) multiple testing correction. Samples with *p* values less than 0.05 (*) and 0.01 (**) are indicated. n.s., non-significant difference (*p* > 0.05)

We also tested if guides based on the commonly used CRISPR-Cas9 system could similarly induce GIGS. Two major differences between Cas13 and Cas9 guides is the endogenous crRNA sequence required for interacting with the corresponding protein (i.e., DR versus trans-activating CRISPR RNA), and the length of the target guide sequence. For Cas13, the crRNA commonly contain 24 to 28 nt corresponding to the target nucleic acid, while Cas9 guides target a sequence of 20 nt. To understand how these differences might impact the elicitation of GIGS, we used the Cas13 single-guide (s-guide 2) that caused a slight yellowing in the leaf and reduced *PDS* mRNA levels (Fig. 1d,e), to design a corresponding 28 nt Cas9 sgRNA (Fig. 6a, sgRNA 2). When the Cas9 designed sgRNAs were delivered by TRV, we observed subtle yellowing in the leaves expressing the 28-nt target sequence, similar to that produced by the Cas13 crRNA design, while TRV expressing the NT-guide did not cause a visible phenotype (Fig. 6b). Importantly, a control Cas9 sgRNA of 28 nt containing 50% mismatches to the PDS sequence showed no yellowing, indicating that the subtle phenotype caused by the Cas9 28-nt guide was specific (Fig. 6b and Additional file 1: Fig. S13 for whole plant images). These visible phenotypes were corroborated by SPAD meter readings that indicated a 28% reduction in chlorophyll content for the Cas9 28-nt guide compared to the control NT-guide, similar to the reduction observed for the Cas13 designed s-guide (Fig. 6c). Molecular quantification indicated significant but variable *PDS* transcript reduction compared to the NT-guide and the 50% mismatch sgRNA controls (Fig. 6d). To test the effect of the spacer guide length, we removed 8 nt from both the 5′ and 3′ target sequence of sgRNA 2 (Fig. 6e). When these two 20 nt Cas9 sgRNA were expressed in TRV, we did not observe the same leaf discoloration compared to that elicited by the Cas9 sgRNA of 28 nt (Fig. 6f, Additional file 1: Fig. S14 for whole plant images). We also did not see a reduction in chlorophyll content or PDS transcript levels (Fig. 6 g,h). These results show that GIGS can be elicited from both the Cas13 and Cas9 designed guides, but that there is a length requirement of the guide spacer sequence. Guides of 20 nt do not appear to elicit GIGS.

## Discussion

The rapid pace of biotechnological innovation for trait manipulation is advancing science and has incredible potential to benefit society. CRISPR-based approaches for RNA manipulation offer new approaches for trait manipulation, but they are currently less well understood compared to DNA-targeting CRISPR. Through the course of our work to develop Cas13 for use in plants, we unexpectedly discovered that the guide crRNA designed for the Cas13a system can reduce viral and endogenous RNA in the absence of the Cas13 protein (i.e., GIGS). There is a question of why this was not previously reported in plants. One explanation is that previous reports of Cas13 function in plants, and other systems have not included a guide-alone control (e.g., stable transgenic line expressing guide crRNA alone) such as the experiments described for stable transgenic rice [17], rice protoplasts [15], and experiments in animal systems [15, 44, 45]. One experiment did test for guide crRNA-alone effects against TuMV in *N. benthamiana*, but reported no impact on viral accumulation [16]. The report only included visible assessment, but no further molecular characterization such as quantifying the level of TuMV or confirming expression of the crRNA, and therefore the data are not conclusive, and the effect of GIGS may have gone unnoticed. Another report in *N. benthamiana* testing Cas13 variants also expressed guide-alone crRNA targeting the tobacco mosaic virus and no GIGS phenotype was reported [18]. The experiment did not include data confirming expression of the crRNA, which could explain the difference, or the discrepancy may be due to other technical differences.

An important distinction for the experiments reported here is our use of multi-guide crRNA in the absence of Cas13. To our knowledge, this control has never been reported in any eukaryotic system to date. Our results suggest that multi-guides in the absence of Cas13 produce substantially more target RNA reduction compared to single-guides alone. Further research is needed to replicate this effect and understand why targeting discontinuous regions produce significantly more RNA reduction. Our extensive characterization of the GIGS phenomena in *N. benthamiana*, demonstration in tomato, verification in stably transformed *A. thaliana*, and evidence provided for a Cas9 designed crRNA collectively show that guides cause target mRNA reduction on their own. Our results show this phenomenon is not dependent on a specific aspect of Cas13 crRNA, as our modified multi-guides lacking bacterial sequence successfully caused target transcript reduction. Likewise, target transcript reduction can be elicited using the Cas9 sgRNA design. However, our results indicate a length requirement, given that the 20 nt targeting sequence failed to elicit silencing. Our results in three plant species are consistent with the report of Cas13-independent transcript silencing in mosquito [43]. We posit that the findings described in mosquito represent the same GIGS phenomena reported here, which suggests that GIGS functions broadly across eukaryotes.

We found that GIGS elicits the production of sRNA with sequence corresponding to the targeted mRNA. Interestingly, multi-guide crRNA stimulated more sRNA production than single-guides, with the majority of sRNA corresponding to the crRNA target sequence, but we also found secondary sRNA targeting intervening regions with sequence not expressed in the crRNA. Given that sRNA are a hallmark of RNAi, it is likely that GIGS functions through endogenous components of RNAi. We note that expressing crRNA through TRV can elicit VIGS, and more generally, expressing exogenous genes can result in post-transcriptional gene silencing (PTGS) [46, 47]. Further research is needed to understand how the crRNA, and specifically the multi-guides described here, elicit target mRNA reduction. This may proceed in the same manner as VIGS or PTGS, or it may involve an altered mechanism of multi-guide paired mRNA processing. Our results show that sequence mismatches at positions 10,11 relative to the 5′ crRNA guide sequence abolished the observed GIGS phenotypes and nearly eliminated target mRNA reduction. We infer these results to show that GIGS is dependent on the endonuclease activity of Argonaute. Interestingly, for Cas13 based crRNA to associate with AGO, it is likely they would first require processing. One possibility for the biogenesis of siRNA from a crRNA could be the processing of the crRNA-mRNA duplex. This could be carried out by one or more Dicer or Dicer-like endogenous ribonuclease III (RNase III) enzyme(s) [48]. While Dicer is conserved across eukaryotes, the gene family has differentially expanded, with a single copy present in vertebrates, two copies present in insects, and up to four Dicers in plants [49, 50]. It is possible that the duplication and diversification of the Dicer superfamily across eukaryotes will affect their competence for GIGS. Differences in Dicer substrate processing have been documented in eukaryotes [51, 52], and further mechanistic understanding is needed for multi-guide crRNA-mRNA processing. Aside from GIGS, it will also be important to determine if Cas13-mediated mRNA cleavage products interact with RNAi machinery to create feedback between the two RNA degradation systems.

## Conclusions

New technological approaches are needed to overcome limitations related to genome editing, particularly for genes that have pleiotropic effects or substantial spatiotemporal regulation. The work presented here suggests that GIGS can achieve target RNA silencing using a guide sequence that is shorter than conventional hairpin and antisense constructs used in plants [46, 47]. This property could be particularly helpful in achieving precision biotech by constructing compact multigene silencing cassettes, which would significantly expand the capabilities of user defined RNA reduction schemes. In principle, multi-guide multi-target silencing could afford a higher target specificity compared to multigene RNAi given the significantly shorter expressed sequences, while avoiding the need to express a Cas13 transgene. Thus, GIGS-based transcriptome engineering could provide a flexible *cis*-genic approach for plant biotechnology.

## Materials and methods

### Designing CRISPR-Cas13a machinery for in planta expression

To develop prokaryotic CRISPR-Cas13a machinery as a platform for in planta transcript silencing, sequences of LbuCas13a and LbaCas13a effectors were *N. benthamiana* codon optimized along with 3x-FLAG tag or 3x-HA tag at the N-terminus, and custom synthesized (Genscript, Piscataway, NJ) (Additional file 2: Table S1). These fragments were assembled using HiFi DNA assembly (New England Biolabs, Ipswich, MA). The integrity of the constructs was confirmed by Sanger sequencing (Genewiz, South Plainfield, NJ).

Turnip mosaic virus engineered to express GFP (TuMV-GFP) [20] and the endogenous phytoene desaturase (*PDS*) gene were selected as targets for CRISPR-Cas13a interference. For crRNA designs, Lba- or LbuCas13a-specific direct repeats with 28 nucleotide spacer sequences complementary to the target were expressed by the *Arabidopsis thaliana* U6 promoter (Additional file 2 Table S2). For TuMV targeting, three single crRNAs guides (s-guide) targeting different regions of TuMV namely 5′ untranslated region (5′ UTR), Helper component Proteinase (HcPro), viral genome linked protein (Vpg), and a multi-guide crRNA containing aforementioned individual crRNAs in an array were designed and constructed (Fig. 1 and Additional file 2 Table S3). Similar to TuMV, the *PDS* transcript was targeted using three single-guide crRNA namely, s-guide 1, s-guide 2, and s-guide 3, and a multi-guide crRNA containing the three single-guides (Additional file 2 Tables S3 and S4).

### Cloning of CRISPR-Cas13a machinery

A backbone harboring AtU6 promoter sequence with one Lbu- or Lba-specific direct repeat sequence and *Bsa*I Golden Gate site was custom synthesized (IDT, Coralville, IA) for expressing crRNAs. This backbone was cloned into entry vector *pENTR* (Thermo Scientific, Waltham MA) using Topo cloning. Spacer sequences were ordered as oligos and cloned using *Bsa*I Golden Gate site. Gateway assembly (Invitrogen) was used to clone the promoter and crRNA cassette into the destination vector *pGWB413* containing or lacking Cas13a effector (Additional file 2 Table S1).

### Cloning crRNA for TRV systemic delivery

For systemic expression of crRNA using TRV, pea early browning virus (PEBV) promoter sequence with LbuCas13a-specific direct repeat and *Bsa*I Golden gate site were custom synthesized (IDT, Coralville, IA) and cloned into Gateway entry vector *PCR8* (Additional file 2 Table S1). Three single-guide and multi-guide crRNA sequences targeting *NbPDS*, and a multi-guide crRNA targeting *SlPDS* were ordered as oligos and cloned using Golden gate assembly (Additional file 2 Table S5). We constructed modified Cas13a crRNA multi-guides targeting *PDS* with the following four modifications: (i) stem-loop: replaced LbuDR with Arabidopsis 24 nt miR170 stem-loop, (ii) loop: Arabidopsis miR170 loop (16 nt), (iii) random: a 20 nt randomly generated nucleotide sequence, and (iv) contiguous: no direct repeat between three s-guide crRNA (Additional file 2 Table S4). These four guide designs were constructed to target the luciferase transcript and served as multi-guide non-target controls (Additional file 2 Table S4). *PDS* and NT m-guide oligos were ordered from IDT, annealed using T4PNK ligase, cloned into pTRV2-PEBV backbone, and selected using Kanamycin. Sanger sequence was conducted to confirm *PDS* and NT m-guide crRNA.

To create mismatch guides corresponding to *PDS* multi-guide crRNA, the nucleotide sequence was altered at positions 5–6 bp, 10–11 bp, and 21–22 bp from the 5′ end of each crRNA (Additional file 2 Table S4). A non-targeting crRNA was designed as a negative control. To create the sgRNA2 construct, we assembled the single-guide 2 target sequence with the trans-activating crRNA (tracrRNA). The same strategy was used to construct sgRNA2 [50%mm] in which single-guide 2 crRNA had mismatches at alternating nucleotides. For sgRNA2 [20 nt-5′], 8 nucleotides were removed from 5′ end of single-guide 2 and assembled with tracrRNA. Similarly, 8 nucleotides were removed from 3′ end of single-guide 2 to construct the sgRNA2 [20 nt-3′] (Additional file 2 Table S4). The NT-sgRNA negative control contained the Cas9 tracrRNA sequence and a non-plant target sequence (Additional file 2 Table S4). The cassette harboring PEBV promoter and TuMV, *NbPDS*, or *SlPDS* targeting crRNAs was PCR amplified with primers having *EcoR*I and *Mlu*I restriction sites and cloned into *EcoR*I and *Mlu*I digested *pTRV2* vector (Additional file 2 Table S6).

### Cloning of intron hairpin RNAi (hpRNAi) cassette

For cloning of *PDS* hpRNAi construct, a 503-bp sequence of PDS gene was custom synthesized as sense and antisense arm along with PDK intron sequence with 25 bp overhang complementarity to *pGWB413* vector (Additional file 2 Table S1). All the fragments were assembled using HiFi DNA assembly (New England Biolabs, Ipswich, MA) expressed by the 35S promoter.

### Agroinfiltration of *N. benthamiana* and *Solanum lycopersicum*

*N. benthamiana* plants were grown and maintained in growth chamber at 23 °C with 16-h light and 8-h dark cycle and 70% humidity. Four-week-old plants were used for leaf spot agroinfiltration to test Cas13a interference against TuMV-GFP. Binary constructs harboring Cas13a homologs with or without crRNA (targeting TuMV or *PDS* transcript), TuMV-GFP infectious clone (a gift from Dr. James Carrington) were individually transformed into chemically competent *Agrobacterium tumefaciens* strain GV3101. Single colonies for each construct were inoculated into LB medium with antibiotics and grown overnight at 28 °C. Next day, the cultures were centrifuged and suspended in agroinfiltration buffer (10 mM MgCl_2_, 10 mM MES buffer pH 5.7, and 100 μM acetosyringone) and incubated at ambient temperature for 2–3 h. For TuMV interference assay, *Agrobacterium* cells harboring Cas13a with crRNA targeting TuMV were infiltrated at an OD600 of 1.0 into adaxial side of four-week-old *N. benthamiana* leaves using a 1.0-ml needleless syringe. Two days later, *Agrobacterium* cells harboring TuMV-GFP were infiltrated into the same areas at an OD600 of 0.3. After 5 days, interference activity of Cas13a against the TuMV-GFP was assayed by visualizing GFP in infiltrated leaves under UV light using a hand-held UV lamp (Fisher Scientific, Waltham, MA) and a Nikon camera.

For *PDS* silencing, leaves of four-week-old *N. benthamiana* plants were infiltrated with *Agrobacterium* cultures harboring LbuCas13a with crRNAs targeting *PDS* and leaf samples were collected at 5 days post inoculation. For TRV-mediated crRNA delivery assays, three-week-old *N. benthamiana* plants were used. A single colony of *Agrobacterium* harboring crRNAs targeting *PDS* were inoculated into LB medium with antibiotics and grown overnight at 28 °C. Next day, the cultures were centrifuged and resuspended into infiltration buffer at an OD600 of 0.6. The cultures were incubated at ambient temperature for 2–3 h and infiltrated into *N. benthamiana*. Two upper leaves were collected 2 weeks after TRV infiltration. Control plants infiltrated with TRV expressing an RNAi antisense fragment (Additional file 2 Table S1) [53] were used to help track systemic TRV movement. Infiltration of tomato plants was performed similarly to *N. benthamiana* except that *Agrobacterium* cells were resuspended into infiltration buffer at an OD600 of 2.0. For tomato *PDS* silencing, an antisense fragment was cloned into TRV (Additional file 2 Table S1) [21]. The cultures were incubated at ambient temperature for 2–3 h and infiltrated into 3-week-old tomato plants. Two weeks after TRV infiltration, a Minolta Chlorophyll Meter SPAD-502DL was used to measure the chlorophyll content in lower leaves, and leaf punches were collected from the photobleached area for quantifying *PDS* expression.

### RNA isolation, cDNA synthesis, qRT-PCR, and northern blotting

Total RNA was isolated from Agro-infiltrated leaf samples and upper leaf tissue following systemic TRV movement using Trizol (Ambion) [54]. For first-strand cDNA synthesis, DNase treated 1 μg total RNA was reverse transcribed using either random hexamers or oligo(dT20) and SuperScript II reverse transcriptase (Thermo Fisher Scientific) according to the manufacturer’s instructions. Quantitative PCR was performed using SYBR Select Master Mix (Applied Biosystem) and gene-specific primers (Additional file 2 Table S6) for *PDS* and TuMV. *EF1*α gene was used as internal house-keeping reference for *PDS* and TuMV qRT-PCR [55]. The experiments were repeated three times with three biological and two technical replicates. Relative expression values were plotted using ggplot2 in R [56, 57]. For detection of *PDS* transcript, 20 μg of total RNA was separated on a denaturing 1.2% agarose gel and blotted on a Hybond-N+ (Roche) membrane. RNA was crosslinked using UV light and hybridized with a DIG labeled probe (PCR DIG probe synthesis kit, Sigma). For detection of LbuCas13a the membrane was stripped and probed with DIG labeled Cas13a-specific probe and signal detected on a Licor Odyssey imaging system (LI-COR Bioscience, Lincoln, NE).

### Real-time quantification of *PDS* and TuMV transcripts using nanocounting technology

For direct RNA quantification of *PDS* and TuMV transcripts using NanoString technology, we collected sequence data for different *N. benthamiana* genes including *PDS*, three house-keeping genes for normalization (*PP2aa2*, *EF1α*, *RPL23a*), LbuCas13a, HCPro, and coat protein (Additional file 2 Table S7). The sequence information was utilized to design two probes for each target gene. Total RNA samples (300 ng total RNA) and probe master mix were supplied to the Huntsman Cancer Institute, University of Utah for Nanostring quantification following manufacturer specifications. The nanocounting data was analyzed using the nSolver software.

### Western blotting

For western blotting, total protein was isolated from *Agrobacterium* infiltrated leaves using extraction buffer (50 mM Tris-Cl, 1% β-Mercaptoethanol, and protease inhibitor cocktail (Roche, Basel, Switzerland)). Total proteins were boiled with loading buffer (100 mM Tris-Cl, 20% Glycerol, 4% SDS, 10% β-Mercaptoethanol, and 0.2 mg/ml bromophenol blue) and resolved on 12% SDS-PAGE gel. The proteins were transferred from SDS-PAGE gel to PVDF membrane (GE healthcare, Chicago, IL). Membrane blocking and antibody incubations were performed using iBind western device (Thermo Fisher Scientific, Waltham, MA) according to the instrument manual. Finally, the membrane was treated with ECL Select western blotting detection reagent (GE healthcare, Chicago, IL) and signal was detected with Licor Odyssey imaging system (LI-COR Bioscience, Lincoln, NE).

### Small RNA sequencing and analysis

Two separate small RNA sequencing experiments were conducted. For results shown in (Fig. 3a–e), Cas13 and crRNA guides and controls were expressed in *N. benthamiana* leaves using agrobacterium spot infiltration as described. Total RNA was extracted from infiltrated leaves using Trizol following the manufacturer’s guidelines. For results shown in (Fig. 3f–l), crRNA guides and controls were expressed from TRV using agrobacterium infiltration as described. Total RNA was extracted from upper leaves following systemic TRV movement using Trizol. Total RNA samples were sent to the Beijing Genomics Institute (BGI Group, Hong Kong). Twenty-four small RNA libraries were constructed following the DNBseq small RNA library protocol. Briefly, small RNA were isolated from PAGE gel corresponding to size 18–30 nt. Adapters were ligated and first-strand synthesis performed according to DNBseq small RNA library protocol. Libraries were PCR amplified and size selected and sequenced on the DNBseq platform (BGI 1Tech Solutions, Hong Kong, China).

Small RNA reads for both experiments were trimmed [58, 59] and aligned using STAR (v2.7.3a) [60] to a modified version of the *N. benthamiana* genome (v1.0.1 )[61]. The modifications included removing all contigs with less than 70 K nt, adding the coding sequence of LbuCas13a as a contig, and masking one of the two paralogs coding for PDS. The coding sequence for *PDS* on contig Niben101Scf14708, position 12885-21779 (gene23) was masked in order to ensure unique mapping to a single *PDS* locus on contig Niben101Scf01283, position 197129-205076 (gene 2002). Uniquely mapped read counts for the exons were extracted per base pair using samtools (v1.3) [62] and bedtools “coverage” (v2.29.2) [63]. Reads were separately mapped to the corresponding crRNA, hairpin, or antisense sequence using BWA (v0.7.17) [64], and uniquely mapped reads were summarized per base of the expressed silencing transcript. To compare between sequenced samples, mapped reads were normalized to library size (i.e., total uniquely mapped reads per library) using the equation (number of reads mapped at a nucleotide position × (1/number of uniquely mapped reads in library) × 1 M), referred to as counts per million (CPM). The size distribution of uniquely mapped reads were analyzed for 21, 22, and 24 nt sRNA. The average number of uniquely mapped sRNA to the *PDS* transcript was calculated for the duplicate samples for each size class. The proportion of each size class was determined by the equation ((average number of reads per size class / sum of average number of reads per size class) × 100). Analyses were carried out using Python3 (v3.8.2) libraries NumPy (v1.18.1), Pandas (1.0.3), and plotted with Matplotlib (v3.2.1) [65–68]. Processed files, additional information, and the reference genome used for mapping are provided through the GEO [69] Series accession number GSE171980 [70].

### Generating stable transgenic Arabidopsis plants

*TTG1*-targeting three single-guides (guide-1, -2, -3) and a multi-guide crRNA (Additional file 2 Table S8), and non-targeting (NT) oligos were annealed and ligated into *pENTR* backbone containing *Bsa*I Golden gate site. Gateway assembly was used to transfer guide crRNA to *pGWB413* destination vector with or without 3xHA-LbuCas13a. Stable transgenic *Arabidopsis* plants expressing *TTG1* guides with or without LbuCas13a were generated using *Agrobacterium*-mediated floral dip [71]. Similarly, stable *Arabidopsis* controls with a NT crRNA, a 197-bp hairpin construct against *TTG1* (a gift from Dr. Steven Strauss, Additional file 2 Table S1) [72], and no guide transformation control (only 3xFLAG-LbuCas13a) were generated. One month after floral dip, T_1_ seeds were collected and stored at 4 °C.

### Arabidopsis phenotyping

Transformed T_1_ Arabidopsis seedlings were identified using rapid selection protocol [73]. Selection was conducted on ½ MS media with a Kanamycin concentration of 100 μg/ml. Positive transformants (*n* = 36) for each *TTG1* crRNA with or without LbuCas13a and *TTG1* hairpin controls were transferred to soil and grown under optimal conditions. Control Arabidopsis Col-0 plants were germinated on ½ MS media without Kanamycin and transferred to soil. Seventh leaf from ten individual plants for each construct was imaged under a dissecting microscope equipped with a Nikon camera and trichomes were counted using multi-point feature in ImageJ software [74]. For each construct, RNA was extracted from 10th leaf of five individual plants with varying leaf trichomes to quantify *TTG1* expression using qRT-PCR. *AtEF1α* was used as internal house-keeping control for normalizing *TTG1* expression (Additional file 2 Table S6). Selected individual plants for each construct were self-pollinated to collect T_2_ seed. Five technical replicates of each selected plant/line were used for analyzing total flavonoids, in 5 mg seed, using modified aluminum chloride (AlCl_3_) colorimetric method [75]. Total flavonoid content was estimated using the following formula: flavonoids (mg/g) = concentration obtained through quercetin calibration curve × (volume of extract/seed weight).

To determine the inheritance of GIGS and Cas13-mediated gene silencing, 10 T_2_ plants from selected T_1_ lines were transferred to soil after Kanamycin selection. Seventh leaf from 10 individual T_2_ plants was imaged for counting leaf trichomes. Statistical comparisons between the transformation control (no guide) and each selected line was performed. *TTG1* expression in the top rosette leaf from three individual T_2_ plants was analyzed using qRT-PCR. Five individual T_2_ plants for each line were self-pollinated to collect T_3_ seed. Total flavonoid content was analyzed in T_3_ seeds from five independent seed lots (five biological replicates). Similarly, proanthocyanidin content was measured using DMACA-HCl method from three seed lots [76]. Proanthocyanidins were measured at 640 nm and reported as per gram of seed weight. Total flavonoid and proanthocyanidin analyses were repeated twice, the averaged values for each seed lot were used for statistical comparisons. Absorbance of flavonoids and anthocyanin was measured using the Thermo Spectronic 3 UV-Visible Spectrophotometer, while absorbance of proanthocyanidins was measured through Synergy H1 Hybrid Multi-Mode Microplate Reader (Agilent Technologies, Winooski, Vermont).

For leaf anthocyanin quantification, 1-week-old T_3_ seedlings after Kanamycin selection were transferred into ½ MS media + 3% sucrose and subjected to light stress (500 μmol m^− 2^ s^− 1^) for 1 week. In total, 200 mg of leaf tissue was used for quantifying anthocyanin [77]. Anthocyanin analysis was repeated twice with 5 replicates in each batch. Anthocyanin content was calculated by using following formula (absorbance/35,000 × dilution factor × 647 × 1000 per mg of sample extracted (in mg g^−1^ fresh weight)). Representative plantlets following sucrose treatment showing anthocyanin pigmentation were imaged with a dissecting microscope equipped with a Nikon camera. To test *TTG1* expression in T_3_ generation, seventh leaf from three individual plants was analyzed using qRT-PCR. To determine the expression of LbuCas13a, RT-PCR was conducted on cDNA synthesized for qRT-PCR. Western blot analysis with HA-tag antibody was conducted on 1-week-old T_3_ seedlings post Kanamycin selection.

## Supplementary Information

**Additional file 1: Fig S1.** Cas13a mediates efficient virus interference. **Fig S2**. crRNA inhibits TuMV accumulation with and without the Cas13 protein. **Fig S3**. GIGS can function systemically to achieve virus interference. **Fig S4.** Guide crRNA design and target sites for endogenous mRNA reduction by GIGS. **Fig S5**. Endogenous mRNA reduction mediated by Cas13-dependent and GIGS expression. **Fig S6**. Guide targets and experimental design for systemic endogenous mRNA reduction by GIGS. **Fig S7**. Systemic endogenous mRNA reduction by GIGS. **Fig S8**. Cas13-dependent and GIGS T_1_ transformed *A. thaliana* lines display phenotypes consistent with *TTG1* reduction. **Fig S9**. Expression and translation products for Cas13 targeting *TTG1* transgenic *Arabidopsis.* **Fig S10**. Small RNA mapping to the expressed crRNA and hairpin constructs targeting the PDS transcript from spot infiltration experiments. **Fig S11**. Small RNA mapping to the expressed crRNA and hairpin constructs targeting the PDS transcript from TRV expression experiments. **Fig S12**. Guide crRNA with mismatches at base pairs 10,11 do not elicit GIGS. **Fig S13**. Cas9 sgRNA can elicit GIGS photobleaching in *N. benthamiana*. **Fig S14**. Cas9 sgRNA induced GIGS is dependent on at least 28 nt targeting sequence in *N. benthamiana*.

**Additional file 2: Table S1.** Plasmids and gene sequences.**Table S2**. Backbones for cloning and expression of crRNA. **Table S3**. crRNA sequences for targeting of TuMV. **Table S4**. crRNA sequences for targeting of *Nicotiana benthamiana PDS.* **Table S5**. crRNA sequences for targeting of tomato *PDS.* **Table S6**. Oligo sequences used in this study. **Table S7**. Probe sequences for Nano-counting. **Table S8**. crRNA sequences for targeting of *Arabidopsis TTG1.*

**Additional file 3.** Review history.

## Data Availability

Original and processed files for the small RNA sequencing data described in this research have been deposited in NCBI’s Gene Expression Omnibus (GEO) [69] and are accessible through GEO Series accession number GSE171980 [70], and through BioProject PRJNA721612.
