# Supplementary material for: CRISPR guides induce gene silencing in plants in the absence of Cas
Source: Genome Biol. 2022 Jan 3;23:6. doi: 10.1186/s13059-021-02586-7 (PMC8722000; doi:10.1186/s13059-021-02586-7)
Supplement: Supplementary file 1 — Additional file 1: Fig S1. Cas13a mediates efficient virus interference. Fig S2. crRNA inhibits TuMV accumulation with and without the Cas13 protein. Fig S3. GIGS can function systemically to achieve virus interference. Fig S4. Guide crRNA design and target sites for endogenous mRNA reduction by GIGS. Fig S5. Endogenous mRNA reduction mediated by Cas13-dependent and GIGS expression. Fig S6. Guide targets and experimental design for systemic endogenous mRNA reduction by GIGS. Fig S7. Systemic endogenous mRNA reduction by GIGS. Fig S8. Cas13-dependent and GIGS T1 transformed A. thaliana lines display phenotypes consistent with TTG1 reduction. Fig S9. Expression and translation products for Cas13 targeting TTG1 transgenic Arabidopsis. Fig S10. Small RNA mapping to the expressed crRNA and hairpin constructs targeting the PDS transcript from spot infiltration experiments. Fig S11. Small RNA mapping to the expressed crRNA and hairpin constructs targeting the PDS transcript from TRV expression experiments. Fig S12. Guide crRNA with mismatches at base pairs 10,11 do not elicit GIGS. Fig S13. Cas9 sgRNA can elicit GIGS photobleaching in N. benthamiana. Fig S14. Cas9 sgRNA induced GIGS is dependent on at least 28 nt targeting sequence in N. benthamiana. [file 13059_2021_2586_MOESM1_ESM.pdf]

ADDITIONAL FILE 1

CRISPR guides induce gene silencing in plants in the absence of Cas

Veerendra Kumar Sharma [1], Sandeep Marla [1], Wenguang Zheng[1], Divya Mishra [1], Jun Huang [1], Wei Zhang[1], Geoffrey Preston Morris [2], David Edward Cook[1]*

1 Department of Plant Pathology, Kansas State University, Manhattan KS, USA

2 Department of Soil and Crop Science, Colorado State University, Fort Collins CO, USA

*Correspondence (decook@ksu.edu)

Fig S1. Cas13a mediates efficient virus interference.

Fig S2. crRNA inhibits TuMV accumulation with and without the Cas13 protein.

Fig S3. GIGS can function systemically to achieve virus interference.

Fig S4. Guide crRNA design and target sites for endogenous mRNA reduction by GIGS.

Fig S5. Endogenous mRNA reduction mediated by Cas13-dependent and GIGS expression.

Fig S6. Guide targets and experimental design for systemic endogenous mRNA reduction by GIGS.

Fig S7. Systemic endogenous mRNA reduction by GIGS.

Fig S8. Cas13-dependent and GIGS T_1 transformed *A. thaliana* lines display phenotypes consistent with *TTG1* reduction.

Fig S9. Expression and translation products for Cas13 targeting *TTG1* transgenic *Arabidopsis.*

Fig S10. Small RNA mapping to the expressed crRNA and hairpin constructs targeting the PDS transcript from spot infiltration experiments.

Fig S11. Small RNA mapping to the expressed crRNA and hairpin constructs targeting the PDS transcript from TRV expression experiments.

Fig S12. Guide crRNA with mismatches at base pairs 10,11 do not elicit GIGS.

Fig S13. Cas9 sgRNA can elicit GIGS photobleaching in *N. benthamiana*.

Fig S14. Cas9 sgRNA induced GIGS is dependent on at least 28 nt targeting sequence in *N. benthamiana*.

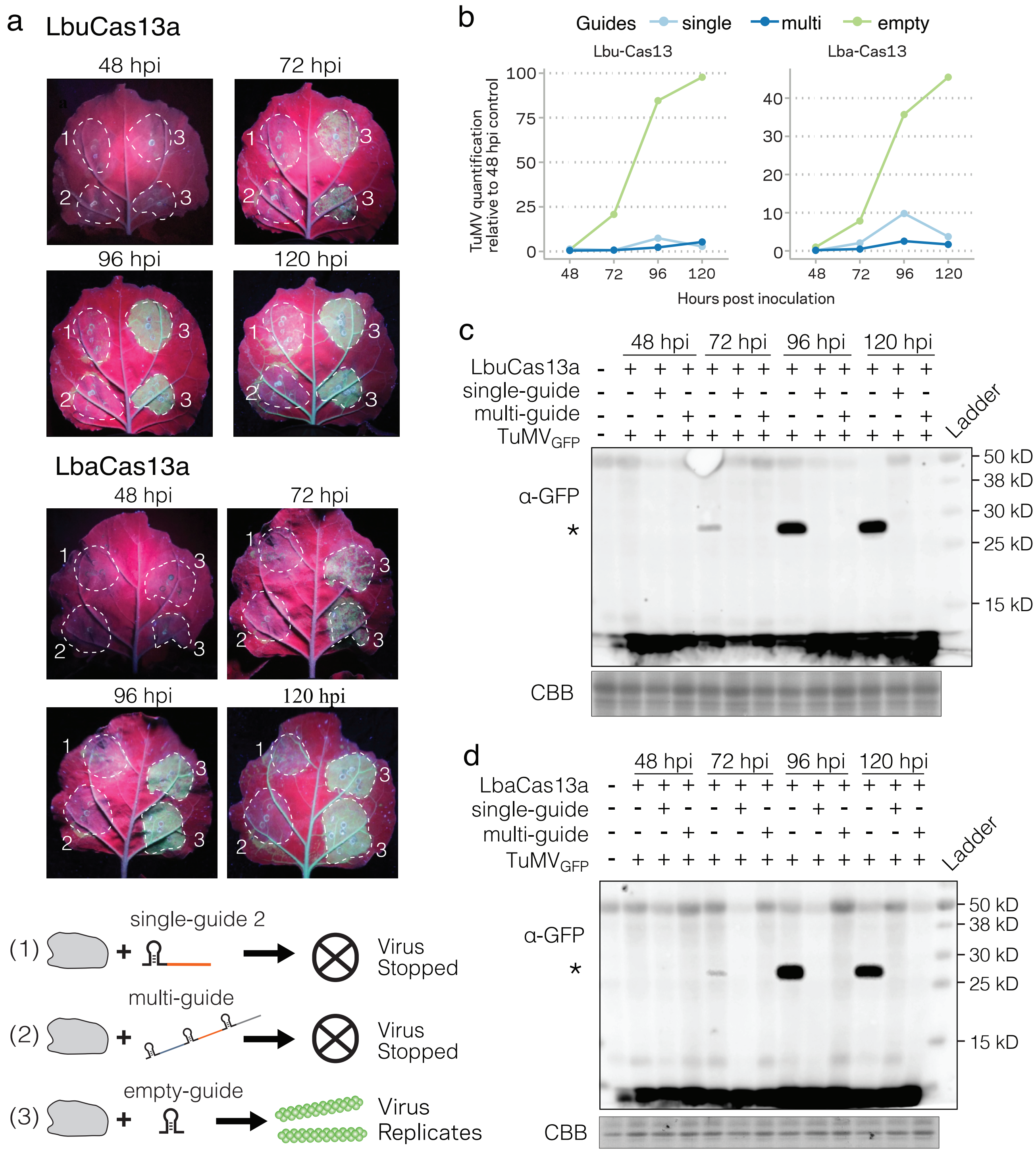

Fig S1. Cas13a mediates efficient virus interference. a, Images of *N. benthamiana* leaves from 48 hours post inoculation (hpi) to 120 hpi shown under UV light to visualize GFP fluorescence. The Cas13 protein (either LbuCas13 or LbaCas13) were expressed with a guide and the TuMV expressing GFP virus as indicated by the schematic diagram and numbering. The areas of agro-infiltration are indicated with white dashed circles. Higher GFP signal results from increased virus accumulation **b,** Quantification of viral genome accumulation using qPCR. TuMV levels were standardized to plant endogenous *EF1α* transcript and three samples were collected per time point. **c,d,** GFP protein accumulation during time series expressing LbuCas13a (c) or LbaCas13a (d). The lanes are labeled above the images for each time point. The anti-GFP (α–GFP) panel shows signal from western blot, with correct sized band indicated with a (*) relative to the ladder shown. Commassie brilliant blue (CBB) stained gel panel shows the loading control.

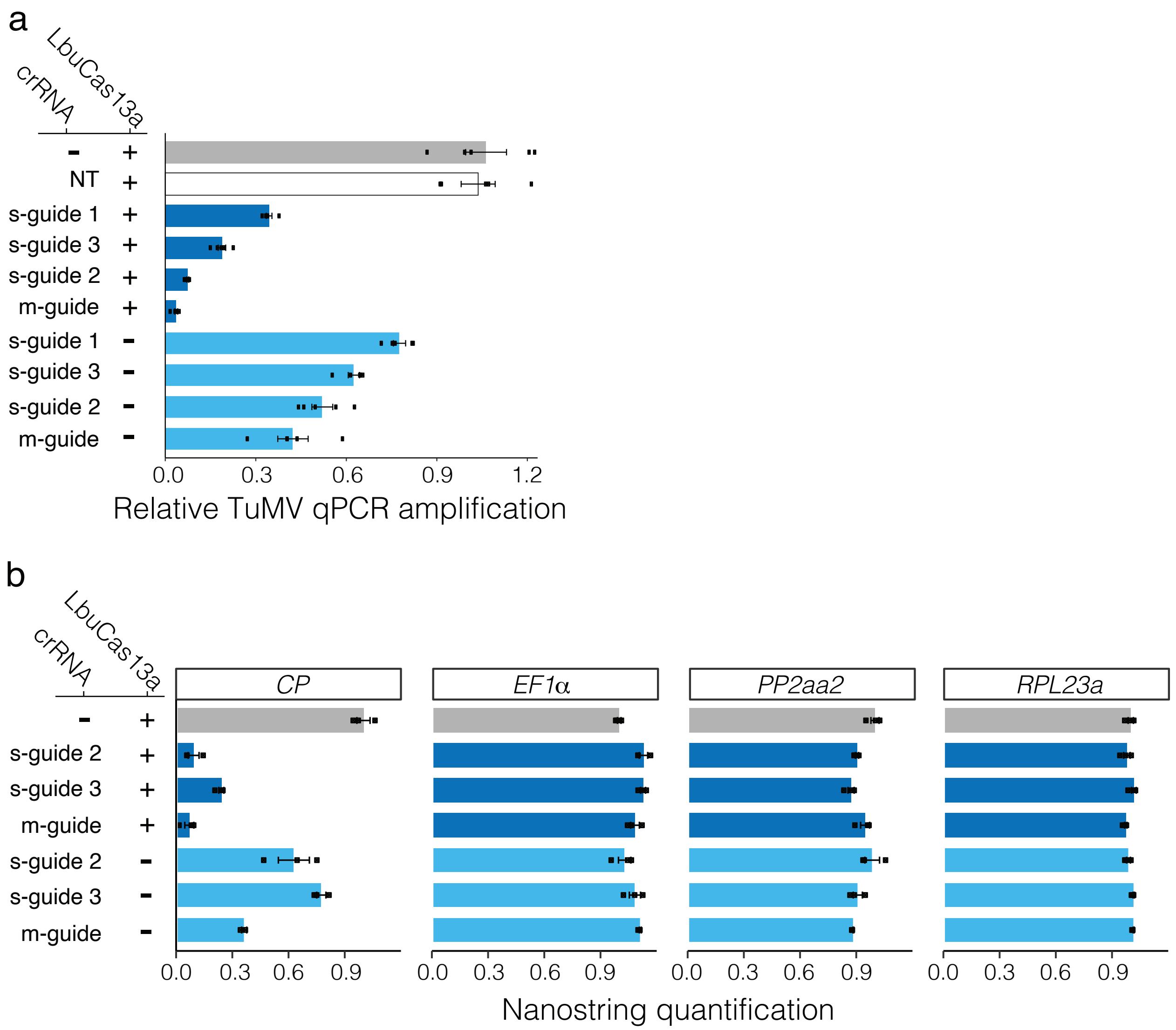

Fig S2. crRNA inhibits TuMV accumulation with and without the Cas13 protein. a, Quantification of TuMV accumulation from *N. benthamina* transient spot expression. Leaves were inoculated with TuMV and a combination of Cas13 and guide crRNA as indicated to the left of the barplot. Individual samples are shown as black dots and the mean is shown as a bar with standard error. TuMV levels were standardized to the plant endogenous *EF1α* transcript and normalized to the Cas13 alone sample. Five samples were collected for each treatment. **b,** Nanostring quantification for using four different probes: TuMV (Coat protein, CP), and three endogenous controls from *N. benthamiana* (*EF1a*, *PP2aa2*, *RPL23a*). Samples expressed either the LbuCas13a protein (+) or no transgene (-), along with no-guide (-), a single-guide (s-guide), or a multi-guide crRNA (m-guide). Three independent samples were analyzed per treatment.

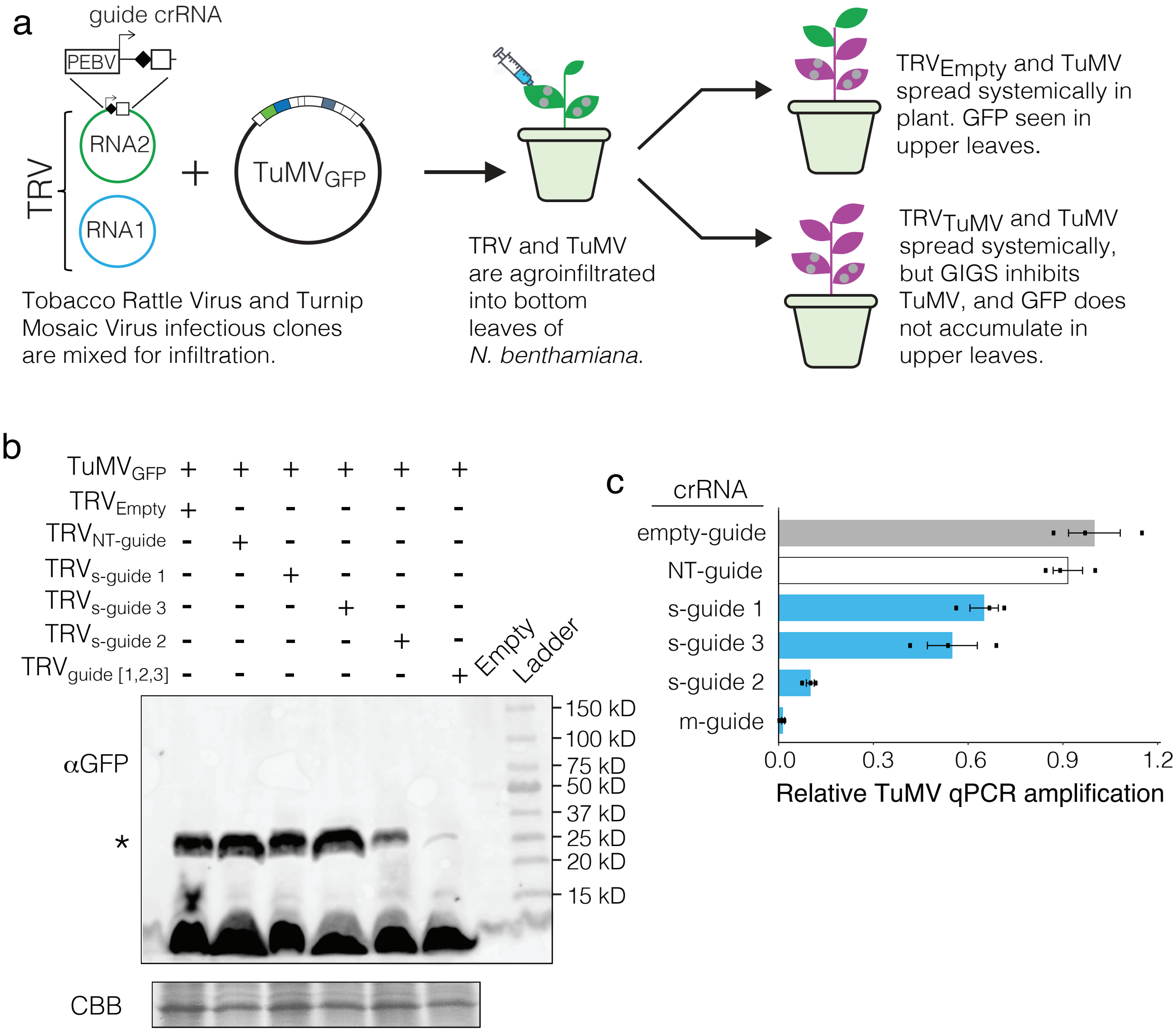

Fig S3. GIGS can function systemically to achieve virus interference. a, Schematic diagram of the TRV expressing guide crRNAs and TuMV expressing GFP. The two infectious clones are mixed and agroinfiltrated into *N. benthamiana*. At 7 days post inoculation, plants are visualized under ultraviolet light. TRV expressing an empty or NT-guide does not target TuMV and GFP accumulates in upper leaves. TRV expressing crRNA targeting TuMV results in GIGS and the plants fail to accumulate GFP. **b,** Upper leaves were checked for GFP accumulation by western blot corresponding to images shown in (Fig. 1d). The GFP antibody panel (αGFP) shows GFP signal, with the GFP specific band indicated with a (*). This is based in the size shown by the ladder. Commassie brilliant blue panel (CBB) shows protein loading. **c,** The TuMV genome was quantified from three independent plants (shown as black points) using qPCR. The mean and standard deviation are shown as barplots. Samples were standardized to the *EF1*α endogenous transcript and normalized to the empty-guide control levels.

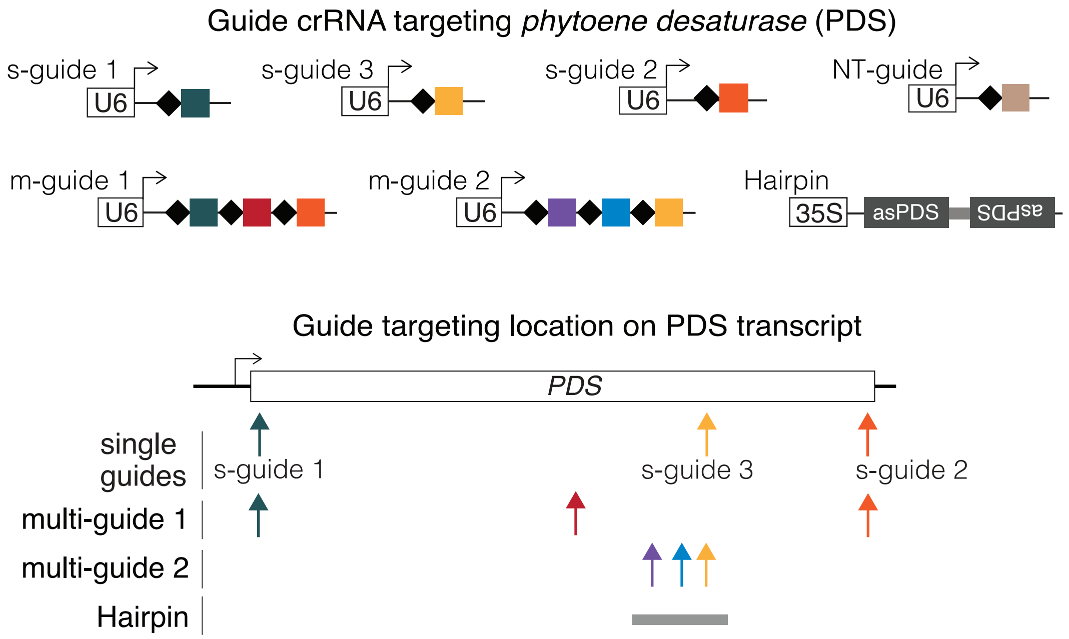

Fig S4. Guide crRNA design and target sites for endogenous mRNA reduction by GIGS.

a, Schematic of single- and multi-guide crRNA (s-guide and m-guide, respectively), along with control non-targeting guides (NT-guide) and RNAi inducing Hairpin. The approximate location where the respective guides are antisense to the PDS transcript (i.e. thier target location) are denoted by filled arrows. The region covered by the hairpin construct is shown as a grey bar.

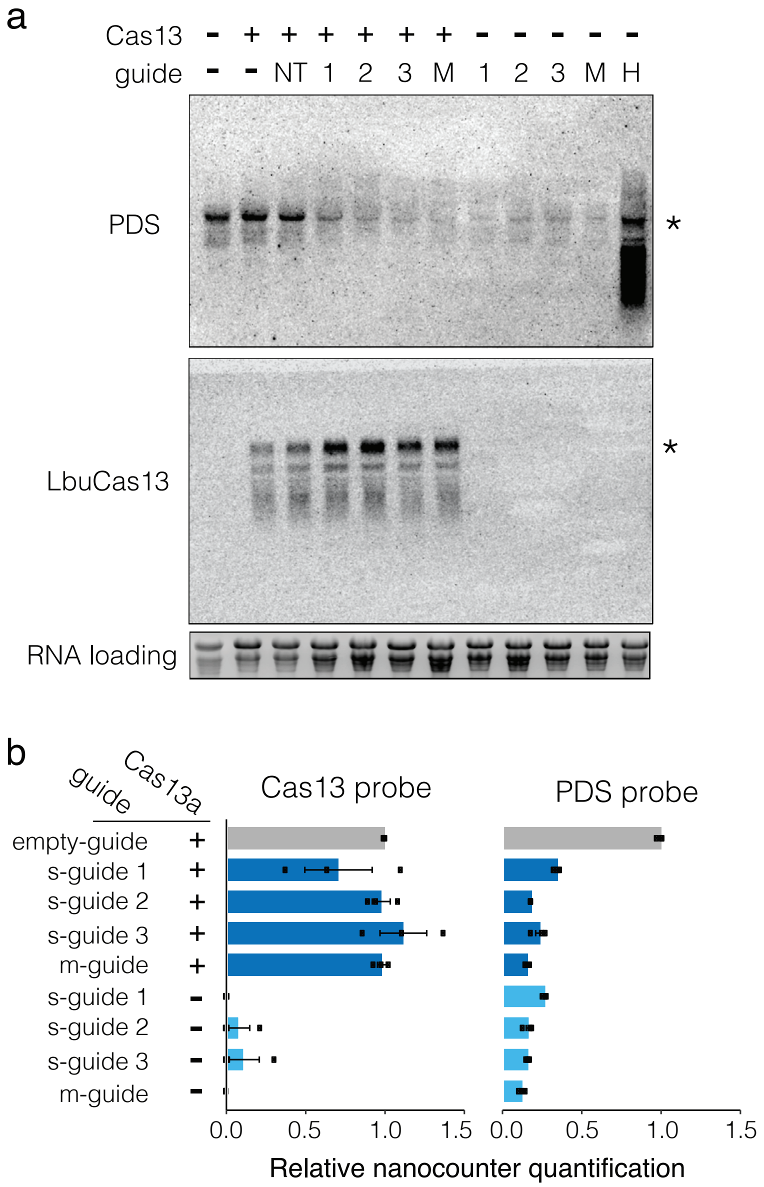

Fig S5. Endogenous mRNA reduction mediated by Cas13-dependent and GIGS expression.

a, Reduction in *PDS* mRNA accumulation as measured by northern blot analysis using a PDS probe (PDS panel). Samples expressing the Cas13a transcript are indicated (Cas13 +) and correspond to signal from the Cas13 probe (LbuCas13 panel). The correct band for each blot is indicated by a (*) based on size. Samples expressed no guide (-), a non-target guide (NT), one of three single-guides (1,2, or 3), a multi-guide (M), or a hairpin construct (H). The presence of roughly equal RNA amounts was confirmed by the abundance of ribosomal RNA signal (RNA loading panel). **b,** Quantification directly on RNA samples using nanostring for Cas13 (Cas13 probe) and *phytoene desaturase* (PDS probe) mRNA. The presence (+) or absence (-) of Cas13 expression is indicated to the left, along with the expression of a single-guide (s-guide) or the multi-guide (m-guide).

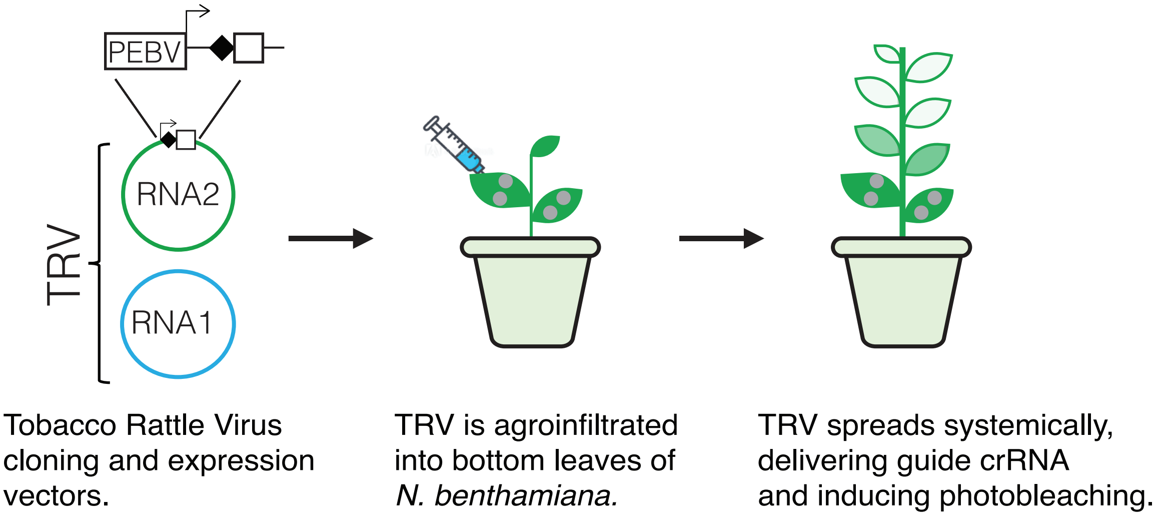

Fig S6. Guide targets and experimental design for systemic endogenous mRNA reduction by GIGS.
Schematic of the TRV expression vector system (RNA1 and 2). RNA2 was engineered to contain the pea early browning virus (PEBV) promoter to express single-, multi-, and NT-guides. An antisense fragment (371 bp) to *PDS* was also inserted into the RNA2 cloning sight to induce RNAi against *PDS*. Infectious clones are agroinfiltrated. TRV moves systemically in the plant, delivering the respective guide crRNA. Those that cause *PDS* mRNA silencing result in a bleaching phenotype (i.e. white sectors) seen on upper leaves.

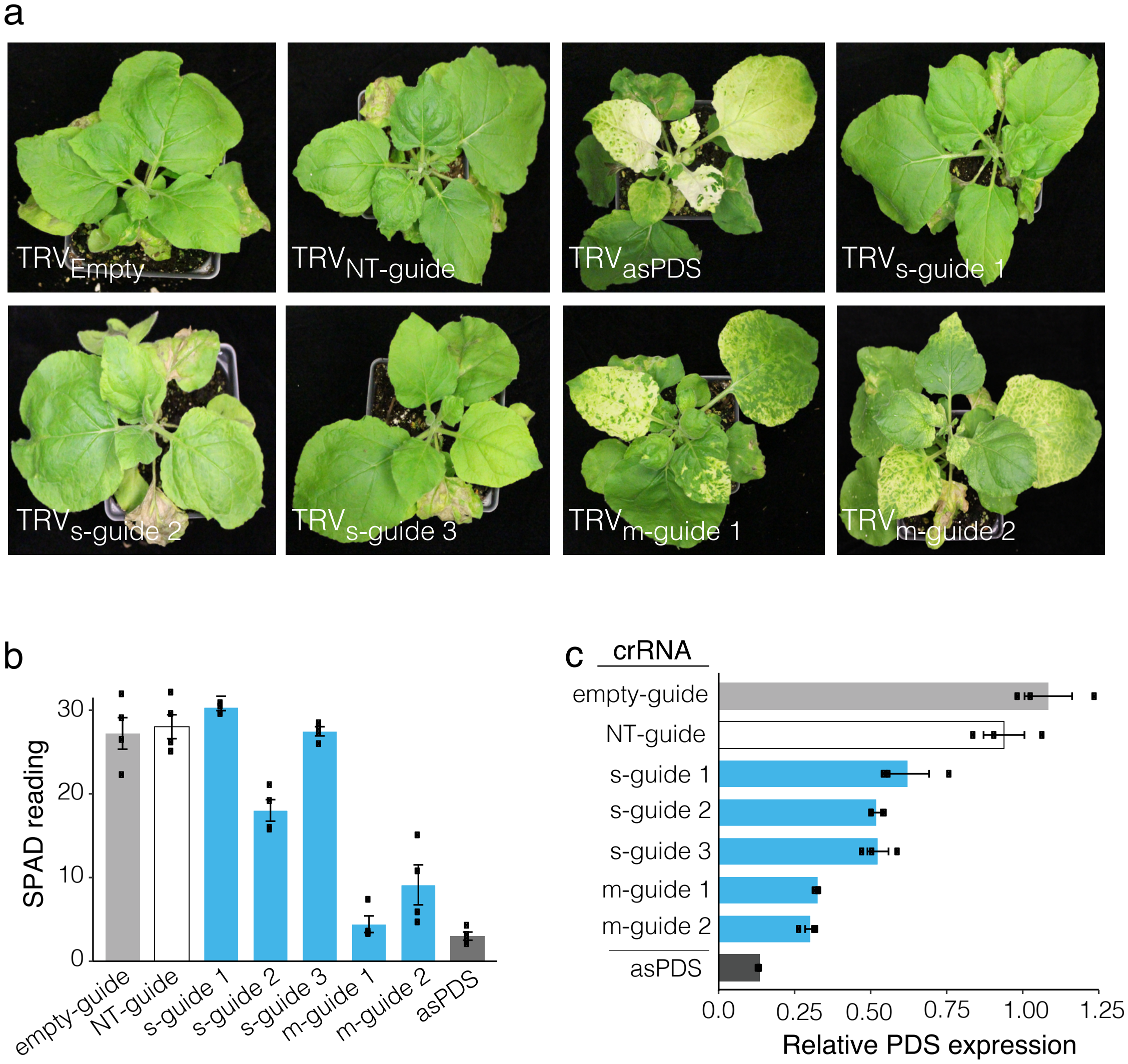

Fig S7. Systemic endogenous mRNA reduction by GIGS.
a, Representative images of *N. benthamiana* plants two weeks post TRV inoculation. TRV moved systemically to the top portion of the plant and expressed guide crRNAs targeting *PDS* or controls. Photobleaching caused by *PDS* mRNA reduction is visible as white or yellow sectors in the upper leaves. Each image is labeled with the guide delivered by TRV. **b,** SPAD meter readings from photobleached areas of leaves. One reading was taken per infiltrated plant. Three independent plants were infiltrated. **c,** The *PDS* transcript was quantified using qPCR from three independent leaves. Data from each sample is shown as a black point with the mean and standard deviation shown as a boxplot. Samples were standardized to the *EF1*α endogenous transcript and normalized to the empty-guide control levels.

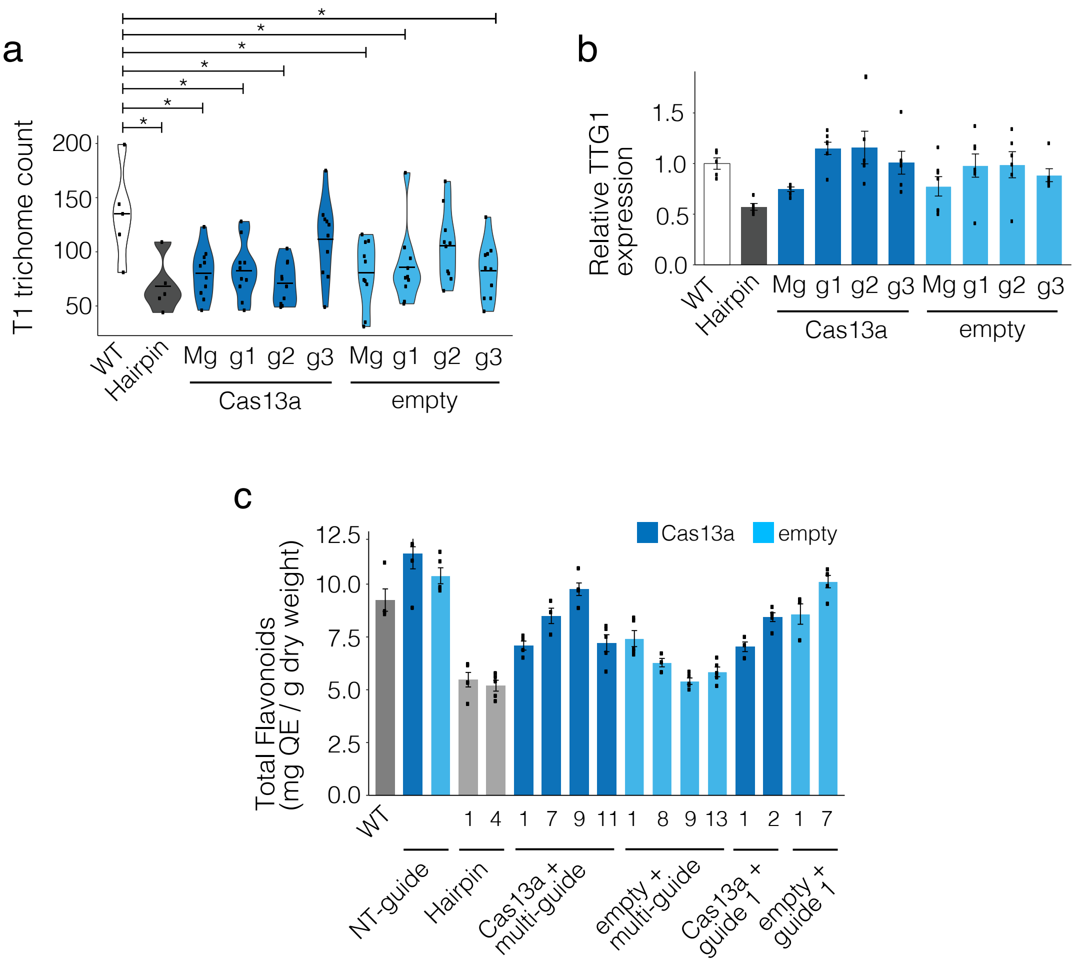

Fig S8. Cas13-dependent and GIGS T_1 transformed *A. thaliana* lines display phenotypes consistent with *TTG1* reduction.

a, Leaf trichome counts from individual T_1 lines shown as black points and the distribution shown as a violin plot. Three single-guides (g1, g2, g3) or a multi-guide (Mg) were transformed into plants with Cas13a (dark blue) or without (light blue). Plants were transformed with a hairpin construct (grey) designed to silence the *TTG1* transcript. Statistical comparisons to the non-transformed control (WT, wild-type) were made as one-sided Mann-Whitney U-test with Benjamini-Hochberg (BH) multiple testing correction. Samples with p-values less than 0.05 (*) are indicated. **b,** Five lines from each transformation group were assessed for *TTG1* transript levels using qPCR. The values were standardized to *AtEF1*α endogenous control and normalized to the WT control. Individual data points are shown as black points and the mean and standard deviation shown as a barplot. **c,** Individual T_1 plants were self fertilized, and five lots of seed from each plant (technical replicates) were analyzed for seed total flavonoid content. Transformants expressing Cas13a are shown in dark blue while those not expressing Cas13a are shown in light blue. Individual data points are shown as black points and the mean and standard deviation shown as barplots. empty, no Cas13a protein; Hairpin, expressing a 197 bp hairpin against *TTG1*.; QE, Quercetin. Numbers indicate the line numbers for the indicated treatments shown below.

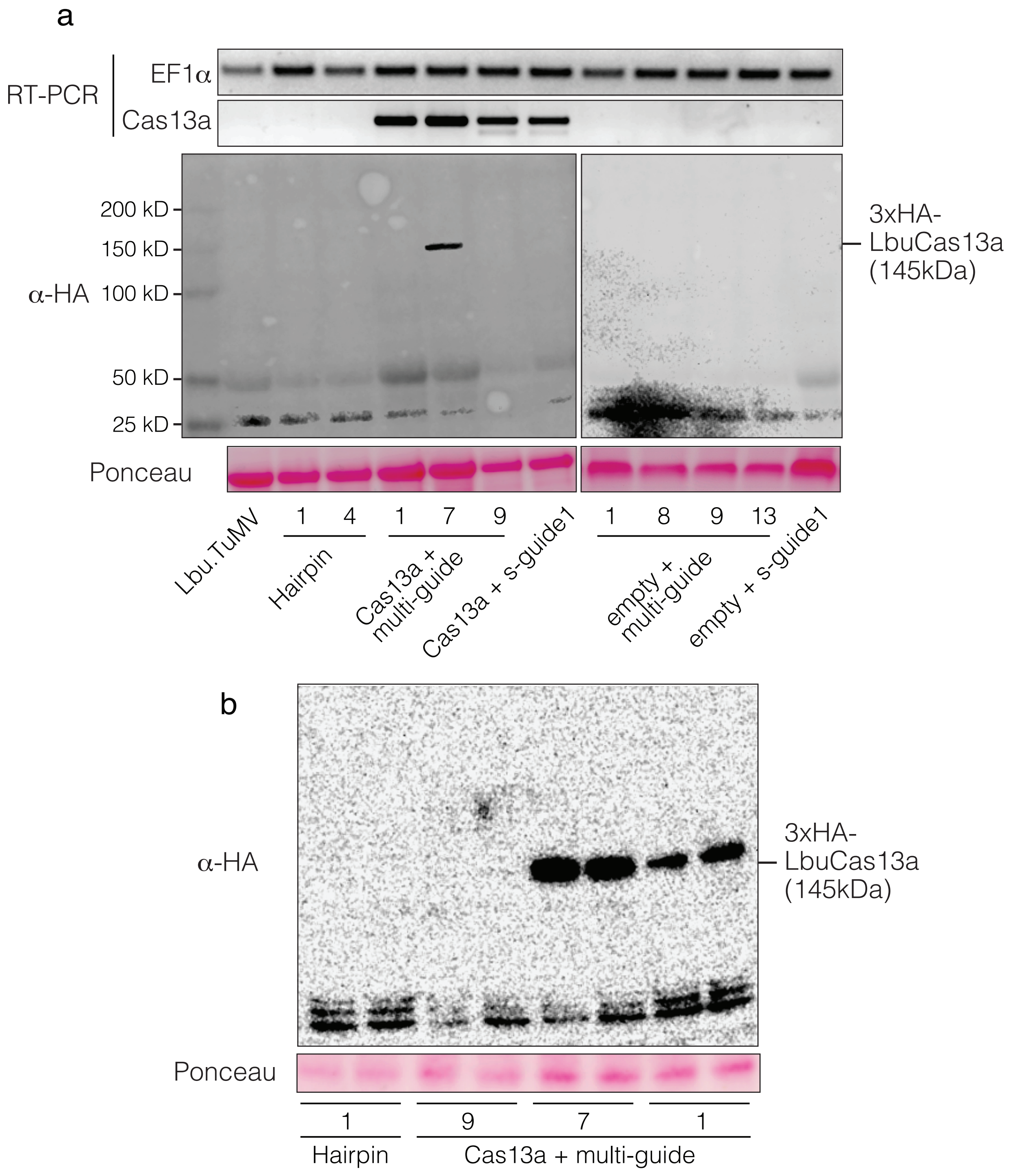

Fig S9. Expression and translation products for Cas13 targeting *TTG1* transgenic *Arabidopsis.*

a, The Cas13 transcript is only detected using reverse-transcription PCR (RT-PCR) in lines transformed with the LbuCas13a transgene (Cas13a panel). The endogenous transcript coding for *EF1*α is shown as a control. The western blot panel using an anti-HA antibody (α-HA) detected a single band corresponding to the size of the 3xHA-LbuCas13a protein (145kDa). Ladder shown on left of blot. Ponceau strain was used as a protein loading control. **b**, Protein was isolated from the same lines used in (a) and re-analyzed by western blot. Higher exposure time detected a single band corresponding to 3xHA-LbuCas13a in line #1 as well as line #7. The results indicate likley differences in protein translation or stability between the different transgenic lines.

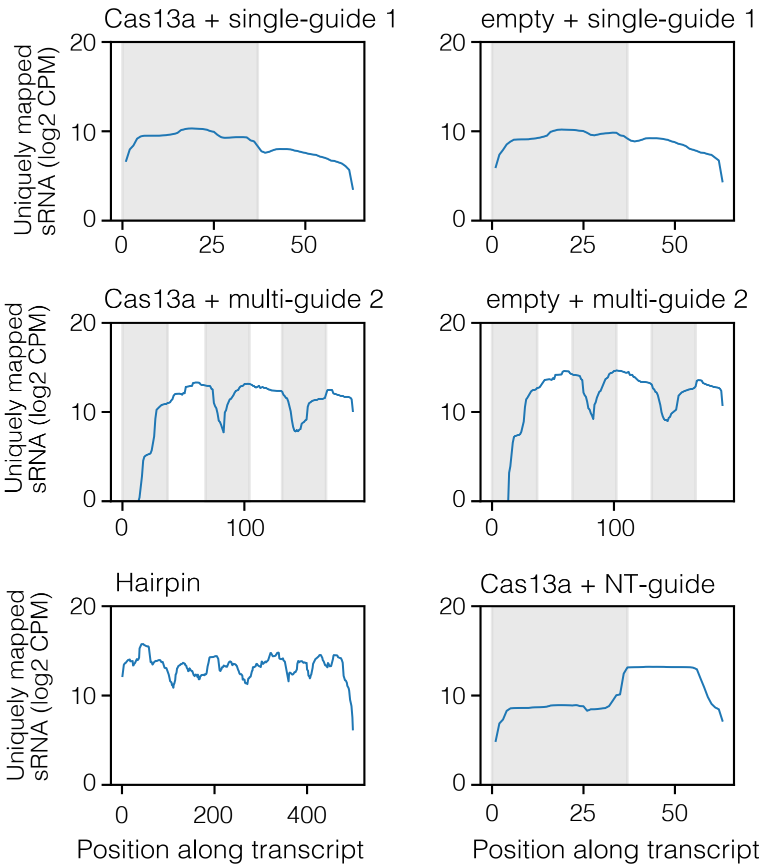

Fig S10. small RNA mapping to the expressed crRNA and hairpin constructs targeting the *PDS* transcript from spot infiltration experiments.

The distribution of uniquely mapped sRNA to the corresponding crRNA are plotted along the coordinates of the crRNA (x-axis). Samples and layout corresponds to main Figure 3. Each sRNA library was independently mapped to the crRNA reference to avoid multi-mapping with the genomic PDS transcript. sRNA reads are summarized per-base and shown as log2 of counts per million (CPM), where one was initially added to the raw counts. The number of uniqley mapped reads per library were used to calculate CPM. The grey boxes highlight the crRNA region(s) that correspond to Cas13 direct repeat (DR) sequence.

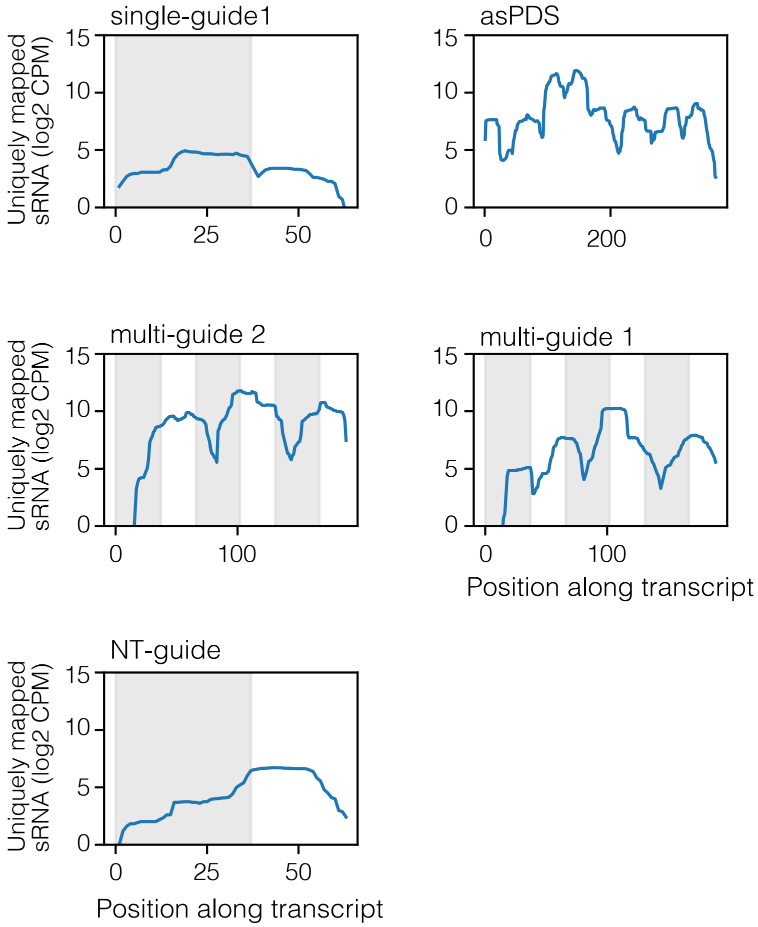

Fig S11. small RNA mapping to the expressed crRNA and hairpin constructs targeting the *PDS* transcript from TRV expression experiments.

The distribution of uniquely mapped sRNA to the corresponding crRNA are plotted along the coordinates of the crRNA (x-axis). Samples and layout corresponds to main Figure 3. Each sRNA library was independently mapped to the crRNA reference to avoid multi-mapping with the genomic PDS transcript. sRNA reads are summarized per-base and shown as log2 of counts per million (CPM), where one was initially added to the raw counts. The number of uniqley mapped reads per library were used to calculate CPM. The grey boxes highlight the crRNA region(s) that correspond to Cas13 direct repeat (DR) sequence.

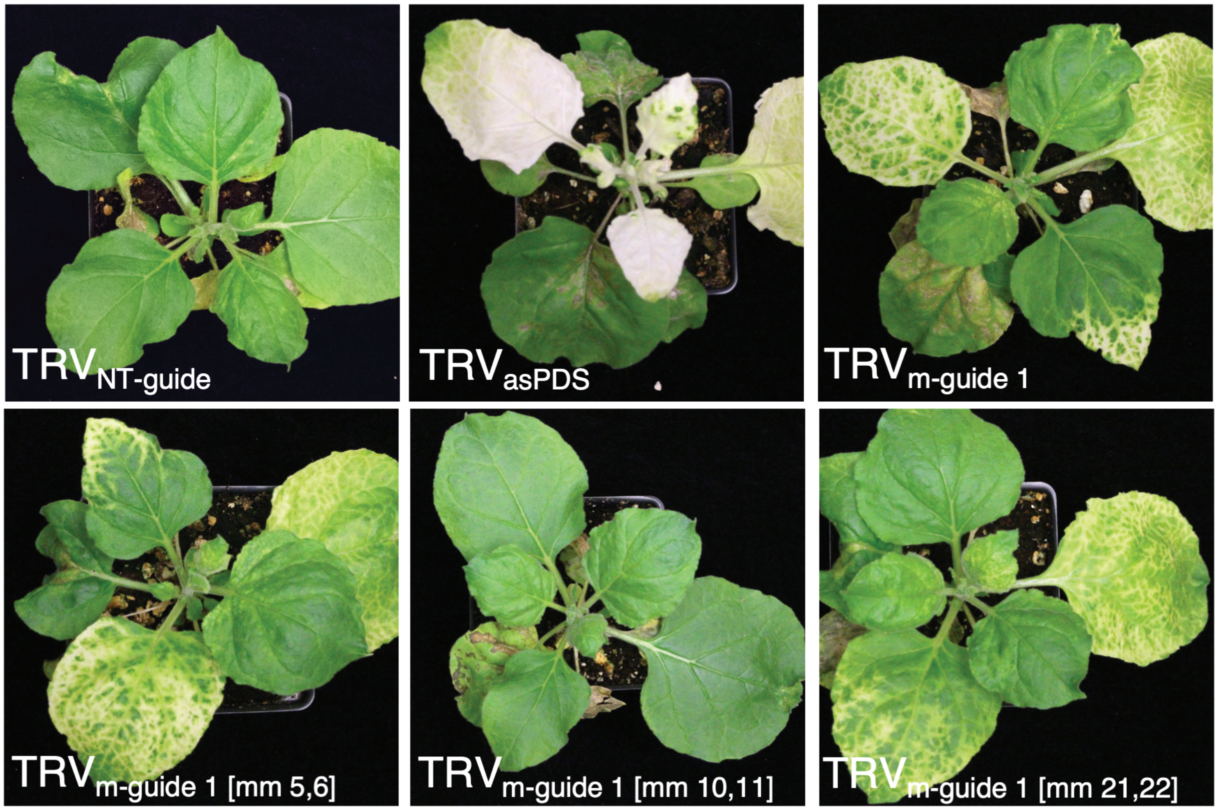

Fig S12. Guide crRNA with mismatches at base pairs 10,11 do not elicit GIGS. Representative images of plants following tobacco rattle virus (TRV) systemic movement. Plants infected with TRV expressing a non-targeting guide crRNA (NT-guide) have a normal green leaf appearance. Plants infected with TRV containing an antisense *PDS* fragment (asPDS) display photobleaching in upper leaves following TRV systemic movement and the triggering of RNAi *PDS* silencing. TRV expressing multi-guide 1 (m-guide 1), multi-guide 1 with mismatches between the target and guide at positions 5 and 6 (m-guide 1[mm 5,6]), or multi-guide 1 with mismatches at positions 21 and 22 (m-guide 1[mm 21,22]) also display photobleaching in upper leaves. Plants inoculated with TRV expressing multi-guide 1 with mismatches between the target and guide at positions 10 and 11 (m-guide 1[mm 10,11]) have the same normal green appearance as the NT-guide samples.

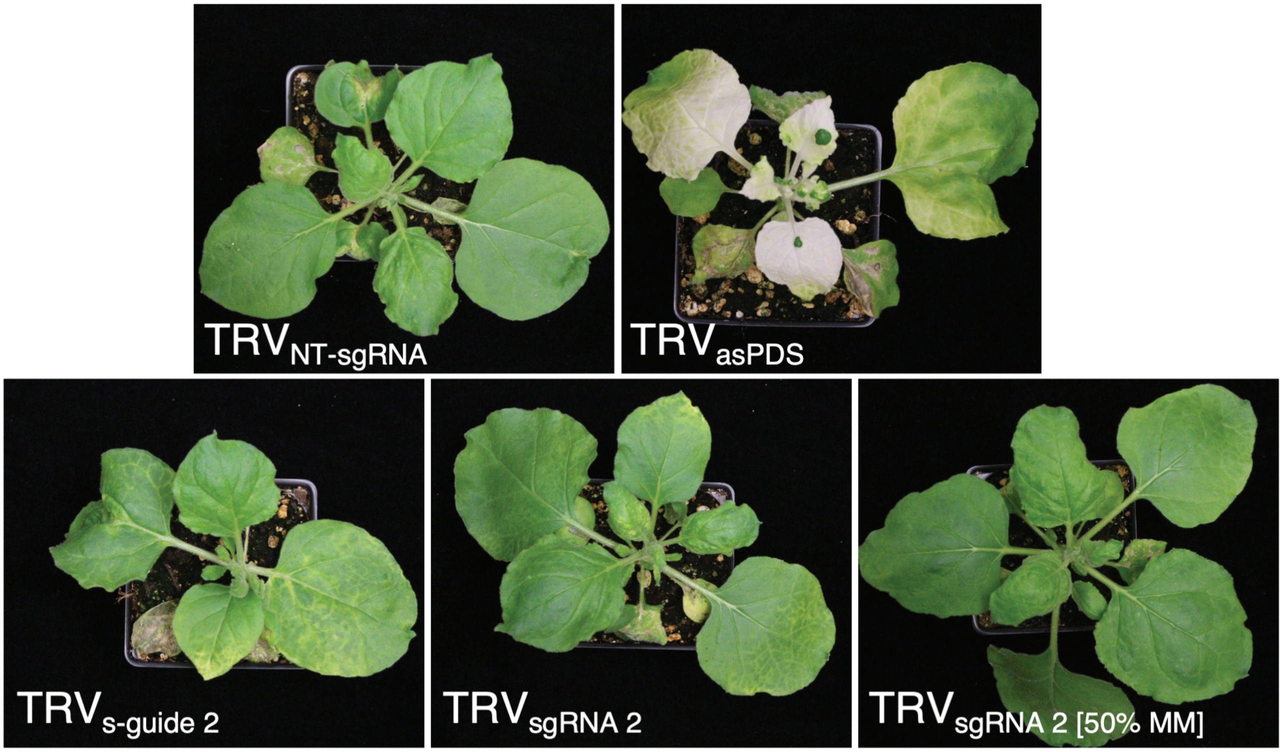

Fig S13. Cas9 sgRNA can elicit GIGS photobleaching in N*. benthamiana.*
Representative whole plant images showing GIGS induced photobleaching. Plants expressing the control non-targeting single guide RNA (NT-sgRNA) from TRV do not display abnormal leaf color. Visible photobleaching is seen from plants expressing TRV containing an antisense *PDS* fragment (asPDS). Clear phenotypic differences in leaf greenness are seen when TRV expressed s-guide 2 (crRNA designed based on Cas13 system) or sgRNA 2 (crRNA designed based on Cas9) targeting *PDS*. These plants show interveinal yellowing. The control Cas9 sgRNA 2 with 50% mismatches to the *PDS* transcript (sgRNA 2[50%mm]) does not display any visible alteration in leaf greenness.

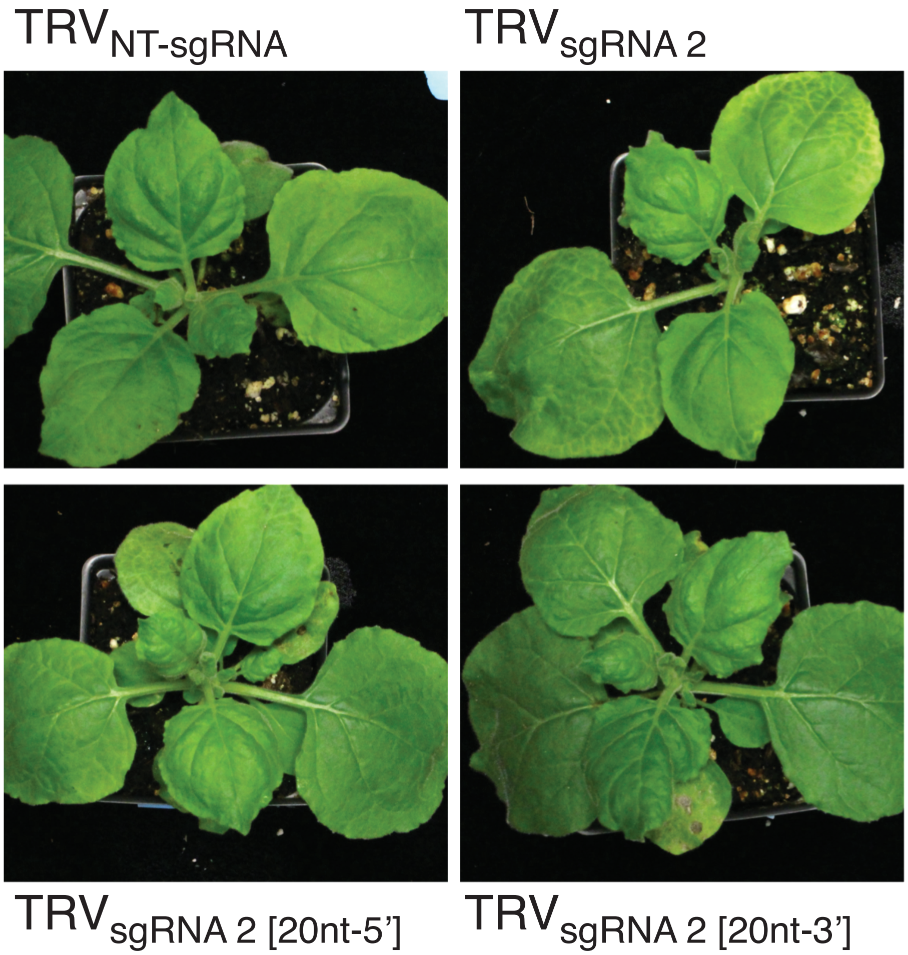

Fig S14. Cas9 sgRNA induced GIGS is dependent on at least a 28 nt targeting sequence in *N. benthamiana.*

Representative whole plant images showing that GIGS induced photobleaching is dependent on crRNA targeting length. Plants expressing the control non-targeting single guide RNA (NT-sgRNA) from TRV did not display abnormal leaf color. A visible loss of leaf greenness is seen when the 28 nt sgRNA 2 construct is expressed. The yellowing is seen in a web or patchy distribution. When 8 of 28 nt of the crRNA sequence targeting PDS are removed from the 5’ or the 3’ end, termed 20nt-5’ and 20nt-3’ respectively, there is no longer a clear visible yellowing as seen for the 28 nt sgRNA 2.
