# Supplementary material for: CRISPR guides induce gene silencing in plants in the absence of Cas
Source: Genome Biol. 2022 Jan 3;23:6. doi: 10.1186/s13059-021-02586-7 (PMC8722000; doi:10.1186/s13059-021-02586-7)
Supplement: Supplementary file 3 — Additional file 3. Review history. [file 13059_2021_2586_MOESM3_ESM.docx]

Review History

**First round of review**

**Reviewer 1**

**Are you able to assess all statistics in the manuscript, including the appropriateness of statistical tests used?** No, I do not feel adequately qualified to assess the statistics.

**Comments to author:**

In this manuscript, the authors describe a phenomenon that crRNAs of Cas13 could induce gene-silencing (GIGS) in the absence of Cas13 to reduce both viral and endogenous RNAs and that GIGS happens in three plants species. GIGS is one kind of exogenous gene induced gene silencing. The manuscript could be greatly improved if some mechanisms of GIGS were provided. It would be usefully to know which part of crRNAs is essential to trigger gene silencing.
There are also some detailed suggestions as well.

1, Line 94-96, Cas13a protein with single or multiple crRNA reduced viral accumulation after inoculation. At 72 hpi, the reduction was obviously observed.

2, Figure 3, the siRNA distribution along crRNAs would be helpfully here.

3, Figure 4, the mutation of mm10,11 is an indirect evidence for AGO1 involvement. The direct evidences should be the GIGS testing with the absence of AGO1 either through ago1 mutants or RNAi knockdown. In addition, the key components of PTGS (DCL4, DCL2, RDR5 etc ) could also be investigated.

**Reviewer 2**

**Are you able to assess all statistics in the manuscript, including the appropriateness of statistical tests used?** No, I do not feel adequately qualified to assess the statistics.

**Comments to author:**

In this research, authors appeared that crRNA alone could induce target RNA degradation. They examined in details and mechanism of guide-induce gene silencing (GIGS) in plants revealed in this study gives us a hint to consider efficient sequence specific RNA reduction approaches. Manuscript is well written but I have a few minor comments.


P14 L387 "we designed a corresponding 28 nt Cas9 sgRNA (Fig.5a, sgRNA1).
Is sgRNA1 a misspelling of sgRNA2?

Authors analyzed mRNA reduction using 28 nt Cas9 sgRNA. However, protospacer of original Cas9, probably SpCas9 is 20 nt. So effects of 20 nt spacer sequence with 78 nt tracrRNA should be analyzed.

**Authors Response**

**Point-by-point responses to the reviewers’ comments:**

Reviewer #1: In this manuscript, the authors describe a phenomenon that crRNAs of Cas13 could induce gene-silencing (GIGS) in the absence of Cas13 to reduce both viral and endogenous RNAs and that GIGS happens in three plants species. GIGS is one kind of exogenous gene induced gene silencing. The manuscript could be greatly improved if some mechanisms of GIGS were provided. It would be usefully to know which part of crRNAs is essential to trigger gene silencing.

We have included new experiments to detail which part of the crRNA is essential to trigger GIGS, including a new figure 5, updated figure 6 and new Supplemental Figure 14. Data in figure 5 demonstrate that modified guide designs, absent of the Cas13 DR, can still elicit GIGS. Updated figure 6 and Supp Fig 14 show that Cas9 crRNA with 28nt guide sequence cause GIGS while 20 nt Cas9 does not (see below for question by reviewer 2). These experiments substantially improve our knowledge for guide design requirements to elicit GIGS.

There are also some detailed suggestions as well.

1, Line 94-96, Cas13a protein with single or multiple crRNA reduced viral accumulation after inoculation. At 72 hpi, the reduction was obviously observed.

The text has been updated to increase readability based on the reviewers comment.

2, Figure 3, the siRNA distribution along crRNAs would be helpfully here.
We mapped the sRNA to the expressed crRNA sequences as requested. The text has been updated and data shown in new Supplemental Figures 10 and 11.

3, Figure 4, the mutation of mm10,11 is an indirect evidence for AGO1 involvement. The direct evidences should be the GIGS testing with the absence of AGO1 either through ago1 mutants or RNAi knockdown. In addition, the key components of PTGS (DCL4, DCL2, RDR5 etc ) could also be investigated.

We agree these are important questions going forward. The most appropriate system to directly identify the genetic requirements is indeed determining the loss of GIGS in various gene deletion mutants or an unbiased screen. Unfortunately these experiments cannot be undertaken in a reasonable time for review. This requires greater than a year of work and substantial funding. We report here the new phenomena of GIGS in plants, and provide significant mechanistic data regarding the creation of siRNA during GIGS, target length requirements, GIGS guide design requirements, heritability, and the loss of GIGS with specific base pair mismatches to the target transcript. This represents a significant amount of new knowledge justifying publication. We have modified the section header in the manuscript to ‘*GIGS RNA reduction may function through Argonaute’* to reflect the indirect nature of the mismatch experiment.

Reviewer #2: In this research, authors appeared that crRNA alone could induce target RNA degradation. They examined in details and mechanism of guide-induce gene silencing (GIGS) in plants revealed in this study gives us a hint to consider efficient sequence specific RNA reduction approaches. Manuscript is well written but I have a few minor comments.

P14 L387 "we designed a corresponding 28 nt Cas9 sgRNA (Fig.5a, sgRNA1).
Is sgRNA1 a misspelling of sgRNA2?

Thank you for point this out, the text should indeed read ‘sgRNA2’ and has been updated.

Authors analyzed mRNA reduction using 28 nt Cas9 sgRNA. However, protospacer of original Cas9, probably SpCas9 is 20 nt. So effects of 20 nt spacer sequence with 78 nt tracrRNA should be analyzed.

We conducted new experiments using a 20 nt spacer sequence for the Cas9 targeting PDS, as the reviewer points out, 20 nt is the common target length used for Cas9 sgRNA. These results have been added to an updated figure 6, Supplemental Figure 14 and the associated text updated. These results help to further define the design requirements for GIGS elicitation and show that a length of at least 28nt is required for GIGS.

**Second round of review**

**Reviewer 1**

This is an improved version and has addressed most of my concerns. However, There are still two questions needs to be addressed by the authors.

1, CrRNAs are delivered through Agrobacterium-transformation or using TRV. It is commonly known that delivery of exogenous genes through Agrobacterium-transformation triggers RNAi though RDR6-mediated PTGS pathway and that delivery of exogenous genes through TRV triggers RNAi though VIGS pathway. Is it possible that crRNA induced RNAi through PTGS or VIGS, given that the dominant 21nt siRNAs and the independence of crRNA DR sequences as well as sgRNAs? Although the answer may not affect the importance of this study, the authors should discuss this possibility.

2, For each comparison in Fig 2(d, e, g, f, i), the compared two sample set need to be clearly labelled. In addition, Mann-Whitney U-test was used for p-value, Benjamini-Hochberg (BH) multiple testing correction was used for FDR. Please correct the Fig. 2 legend what * and ** means.

**Authors Response**

**Point-by-point responses to the reviewers’ comments:**

Reviewer #1:

This is an improved version and has addressed most of my concerns. However, There are still two questions needs to be addressed by the authors.
1, CrRNAs are delivered through Agrobacterium-transformation or using TRV. It is commonly known that delivery of exogenous genes through Agrobacterium-transformation triggers RNAi though RDR6-mediated PTGS pathway and that delivery of exogenous genes through TRV triggers RNAi though VIGS pathway. Is it possible that crRNA induced RNAi through PTGS or VIGS, given that the dominant 21nt siRNAs and the independence of crRNA DR sequences as well as sgRNAs? Although the answer may not affect the importance of this study, the authors should discuss this possibility.

*We added the following text to the discussion: We note that expressing crRNA through TRV can elicit VIGS, and more generally, expressing exogenous genes can result in post-transcriptional gene silencing (PTGS) [46,47]. Further research is needed to understand how the crRNA, and specifically the multi-guides described here, elicit target mRNA reduction. This may proceed in the same manner as VIGS or PTGS, or it may involve an altered mechanism of multi-guide paired mRNA processing.

This statement addresses the reviewers comment and acknowledges that the observed phenomena maybe mechanistically related to VIGS or PTGS or may have further mechanistic distinctions.

2, For each comparison in Fig 2(d, e, g, f, i), the compared two sample set need to be clearly labelled. In addition, Mann-Whitney U-test was used for p-value, Benjamini-Hochberg (BH) multiple testing correction was used for FDR. Please correct the Fig. 2 legend what * and ** means.
*We updated the figure legend as follows: (**d,e,g-i**) Statistical comparisons were made between the transformation control (no guide) and each treatment using a one-sided Mann-Whitney U-test with Benjamini-Hochberg (BH) multiple testing correction. A line is shown above each pair of bars to indicate each pairwise comparison that had a statistically significant difference. This is indicated for comparisons where the adjusted p-value was less than 0.05 (*) and 0.01 (**).

We believe this clearly states that each treatment was compared to the control and that statistical differences at the adjusted p-value level of 0.05 and 0.01 are shown.
